# AGPAT2 interaction with CDP-diacylglycerol synthases promotes the flux of fatty acids through the CDP-diacylglycerol pathway

**DOI:** 10.1038/s41467-021-27279-4

**Published:** 2021-11-25

**Authors:** Hoi Yin Mak, Qian Ouyang, Sergey Tumanov, Jiesi Xu, Ping Rong, Feitong Dong, Sin Man Lam, Xiaowei Wang, Ivan Lukmantara, Ximing Du, Mingming Gao, Andrew J. Brown, Xin Gong, Guanghou Shui, Roland Stocker, Xun Huang, Shuai Chen, Hongyuan Yang

**Affiliations:** 1 School of Biotechnology and Biomolecular Sciences, the University of New South Wales, Sydney, NSW 2052 Australia; 2 MOE Key Laboratory of Model Animal for Disease Study, Model Animal Research Center, School of Medicine, Nanjing University, 210061 Nanjing, China; 3 Heart Research Institute, The University of Sydney, Newtown, NSW 2042 Australia; 4 Faculty of Medicine and Health, The University of Sydney, Sydney, NSW 2006 Australia; 5 Victor Chang Cardiac Research Institute, Darlinghurst, NSW 2010 Australia; 6 State Key Laboratory of Molecular Developmental Biology, Institute of Genetics and Developmental Biology, Chinese Academy of Sciences, 100101 Beijing, China; 7 Department of Biology, Southern University of Science and Technology, 518055 Shenzhen, Guangdong China; 8 Lipidall Technologies Company Limited, 213022 Changzhou, Jiangsu Province China; 9 Laboratory of Lipid Metabolism, Hebei Medical University, 050017 Shijiazhuang, Hebei China; 10 School of Life and Environmental Sciences, The University of Sydney, Sydney, NSW 2006 Australia

**Keywords:** Multienzyme complexes, Mechanisms of disease, Phospholipids, Metabolic syndrome

## Abstract

AGPATs (1-acylglycerol-3-phosphate *O*-acyltransferases) catalyze the acylation of lysophosphatidic acid to form phosphatidic acid (PA), a key step in the glycerol-3-phosphate pathway for the synthesis of phospholipids and triacylglycerols. AGPAT2 is the only AGPAT isoform whose loss-of-function mutations cause a severe form of human congenital generalized lipodystrophy. Paradoxically, AGPAT2 deficiency is known to dramatically increase the level of its product, PA. Here, we find that AGPAT2 deficiency impairs the biogenesis and growth of lipid droplets. We show that AGPAT2 deficiency compromises the stability of CDP-diacylglycerol (DAG) synthases (CDSs) and decreases CDS activity in both cell lines and mouse liver. Moreover, AGPAT2 and CDS1/2 can directly interact and form functional complexes, which promote the metabolism of PA along the CDP-DAG pathway of phospholipid synthesis. Our results provide key insights into the regulation of metabolic flux during lipid synthesis and suggest substrate channelling at a major branch point of the glycerol-3-phosphate pathway.

## Abstract (web summary)

AGPATs (1-acylglycerol-3-phosphate O-acyltransferases) catalyze the acylation of lysophosphatidic acid to form phosphatidic acid (PA), a key step in the synthesis of all glycerolipids. Here, the authors show that AGPAT2 and CDP-DAG synthases (CDS1 and CDS2) form functional complexes that promote further conversion of PA along the CDP-DAG pathway of phospholipid synthesis.

## Introduction

Living organisms need to store energy to survive in an ever-changing environment. For cells, energy is stored in the form of neutral lipids within lipid droplets (LDs), which are evolutionarily conserved organelles found in nearly all organisms^1–3^. Mammals have developed adipocytes and adipose tissue, which specialize in energy storage^4,5^. Adipocytes are derived from mesenchymal stem cells through a process called adipogenesis that is regulated by a transcriptional cascade. Understanding the fundamental mechanisms governing LD formation/growth (cellular lipid storage) and adipogenesis (systemic lipid storage) may provide better treatment strategies against obesity and its associated metabolic disorders.

**Fig. 1:**
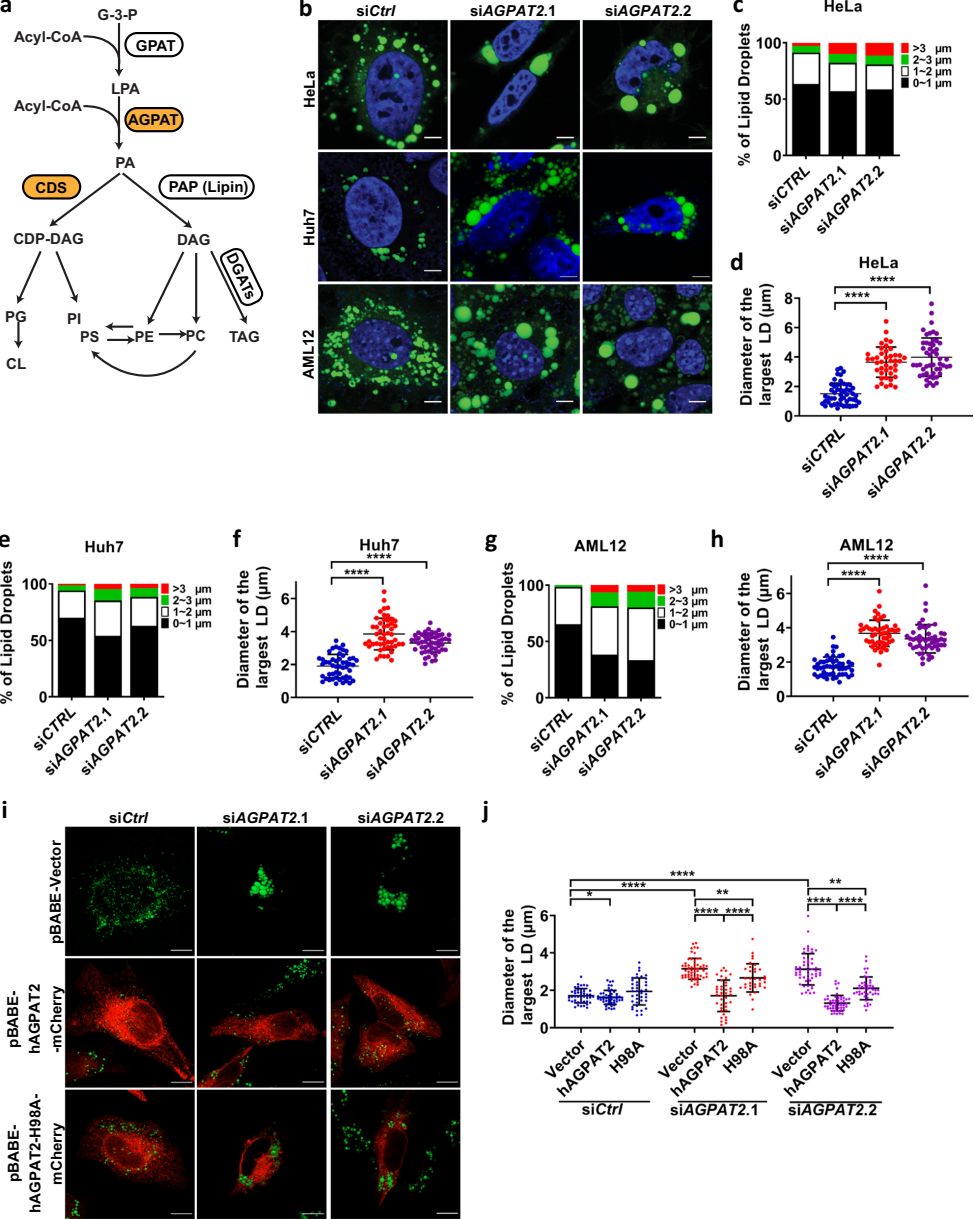
AGPAT2 depletion alters LD morphology. **a** The glycerol-3-phosphate (G-3-P) pathway for the synthesis of phospholipids and triacylglycerols. GPAT glycerol-3-phosphate *O*-acyltransferase, AGPAT 1-acylglycerol-3-phosphate *O*-acyltransferase, LPA lysophosphatidic acid, PA phosphatidic acid, PAP PA phosphatase, CDS CDP-DAG synthase, DAG diacylglycerol, PS phosphatidylserine, PE phosphatidylethanolamine, PC phosphatidylcholine, TAG triacylglycerol, PG phosphatidylglycerol, PI phosphatidylinositol, CL cardiolipin. **b** Confocal imaging of LDs in control and HeLa, Huh7 and AML12 cells treated with *AGPAT2* siRNA. The cells were treated with 20 nM siRNA for 24 h, followed by oleate treatment (400 μM) for 18 h. Blue represents DAPI staining, and green represents BODIPY staining. Bars: 5 μm. **c**, **e**, **g** Bar graphs show LD size distribution in HeLa, Huh7 and AML12 cells. Diameter of all detectable LDs in a cell was measured and represented by red (>3 µm), green (2–3 µm), white (1–2 µm) and black (0–1 µm) (*n* = 42–50 cells examined over 3 biologically independent experiments). **d**, **f**, **h** Quantification of LD diameters in HeLa, Huh7 and AML12 cells. Diameter of the largest LD in a cell was measured (mean ± SD; ****p < 0.0001, one-way ANOVA, *n* = 42–50 cells examined over three biologically independent experiments). **i** Human AGPAT2-mCherry or the catalytic dead mutant H98A was overexpressed in *AGPAT2* knockdown HeLa cells. Cells were treated with 400 μM oleic acid for 18 h. Green represents BODIPY staining and red indicates mCherry expression. Bars = 5 μm. **j** Quantification of LD diameters after overexpressing vector, AGPAT2-mCherry, and AGPAT2-H98A-mCherry in AGPAT2 knockdown HeLa cells. Diameter of the largest LD in a cell was measured (mean ± SD; ****p < 0.0001; **p < 0.01, two-way ANOVA, *n* = 31–59 cells examined over three biologically independent experiments).

The 1-acylglycerol-3-phosphate *O*-acyltransferases (AGPATs), also known as lysophosphatidic acid acyltransferases (LPAATs) are intermediate enzymes in the biosynthesis of phospholipids and triacylglycerols (TAGs) through the glycerol-3-phosphate pathway (Fig. 1a)^6,7^. The first committed step is the acylation of glycerol-3-phosphate to form 1-acylglycerol-3-phosphate (also called lysophosphatidic acid (LPA)). This reaction is catalyzed by glycerol-3-phosphate *O*-acyltransferases (GPATs). AGPATs/LPAATs esterify the *sn*-2 position of LPA to make phosphatidic acid (PA), a critical intermediate that can be dephosphorylated into diacylglycerol (DAG) by PA phosphatases (PAPs, e.g., lipins), or converted into CDP-DAG by CDP-DAG synthases 1 and 2 (CDS1 and 2)^8^. To date, at least 5 putative human AGPAT isoforms have been identified, each encoded by a different gene^7^. Among the AGPAT isoforms, AGPAT2 stands out since null mutations of AGPAT2 are associated with Berardinelli-Seip congenital lipodystrophy type 1 (*BSCL1*)/congenital generalized lipodystrophy, type 1 (*CGL1*), which is characterized by a near complete loss of adipose tissue, early onset of insulin resistance, diabetes, hypertriglyceridemia, and hepatic steatosis^9^. Human AGPAT2 localizes to the endoplasmic reticulum (ER), and is expressed abundantly in adipose tissue, liver and pancreas^10^. Importantly, *Agpat2*^*−/*−^ mice replicate most of the features of human *BSCL1/CGL1*, although the extent of fat loss and the degree of insulin resistance appear more severe in mice than in humans^11^.

Exactly how AGPAT2 deficiency causes lipodystrophy, i.e., *BSCL1*, is unknown^11,12^. While AGPAT2 catalyzes the acylation of LPA to PA, the level of PA was surprisingly and dramatically increased in AGPAT2-deficient cells and liver^11–13^. In this connection, seipin, whose loss-of-function mutations cause *BSCL2*, also regulates the level and distribution of PA^14–18^. PA and cyclic PA have been shown to antagonize PPARγ, a ligand-activated transcription factor essential for adipogenesis^19,20^. Thus, both AGPAT2 and seipin could regulate adipogenesis through modulating the level/distribution of PA. Moreover, PA is the only cone-shaped anionic lipid in a cell^21^. Due to its shape and negative charge, PA’s concentration/distribution can greatly impact membrane function. An increase in local PA content may affect ER surface tension/curvature and alter the biogenesis/growth of LDs^15,17,22^. Indeed, seipin has been established as a key regulator of the initiation and growth of LDs^14,22–24^. If and how AGPAT2 may impact LD formation remains unexplored.

In this work, we set out to investigate LD initiation and growth in AGPAT2-deficient cells. We discover an unexpected link between AGPAT2 and CDS1/2, enzymes that convert PA to CDP-DAG for the synthesis of phospholipids including phosphatidylinositol (PI) and phosphatidylglycerol (PG) (Fig. 1a). Our results unveil a mechanism by which AGPAT2 deficiency may lead to an increase of cellular PA and cause lipodystrophy. More broadly, our results suggest the existence of substrate channelling at a key branch point of the glycerol-3-phosphate pathway for the synthesis of phospholipids and TAGs.

## Results

### AGPAT2 deficiency results in the formation of supersized LDs and a delay in initial LD lipidation

Tagged and overexpressed human AGPAT2 was shown to localize to the ER^10^. However, the localization of endogenous AGPAT2 has not been determined, possibly due to a lack of an appropriate anti-AGPAT2 antibody. We tagged AGPAT2 with super folder GFP (sfGFP) at its genomic locus by CRISPR, and found that AGPAT2–sfGFP colocalized with calnexin, an ER marker (Supplementary Fig. 1a). Interestingly, a portion of AGPAT2 appeared very close to LDs (Supplementary Fig. 1a). The fluorescence signal disappeared after knocking-down AGPAT2 by siRNA, indicating the accuracy of GFP integration (Supplementary Fig. 1a, b).

Given its ER localization and known impact on PA metabolism, AGPAT2 may regulate the formation of LDs. To test this hypothesis, AGPAT2 was knocked down by siRNA in three different cells lines: HeLa, Huh7 and AML12. AGPAT2 deficiency led to the formation of giant LDs (defined as LDs with diameters >2 µm) after prolonged oleate treatment (18 h) in all three cell lines (Fig. 1b–h). The amount of TAG was also significantly increased under AGPAT2 deficiency (Supplementary Fig. 1c). We also knocked out AGPAT2 in these cells by CRISPR, but the knock-out cells appeared very sick (data not shown) and therefore not used. Wild type (WT) AGPAT2, but not a catalytic inactive mutant (H98A)^25^, rescued the LD phenotype when expressed in the knockdown cells (Fig. 1i, j). Moreover, the sizes of LDs were also increased when AGPAT2 was knocked down in 3T3 L1 adipocytes, a professional cell for fat storage (Supplementary Fig. 1d–f). Knocking down AGPAT2 did not seem to impact the viability of 3T3 L1 adipocytes (Supplementary Fig. 1g). To characterize the formation of supersized LDs in greater detail, we examined LD formation over 24 h of oleate treatment. Within 2 h of oleate addition, LDs in AGPAT2-depleted cells were larger than those in control cells, although almost all LDs were <2 µm in diameter (Supplementary Fig. 1h, i). Giant LDs first appeared in AGPAT2 knockdown cells ~8 h after adding oleate and became more prominent later. In contrast, giant LDs were extremely rare in control cells.

**Fig. 2:**
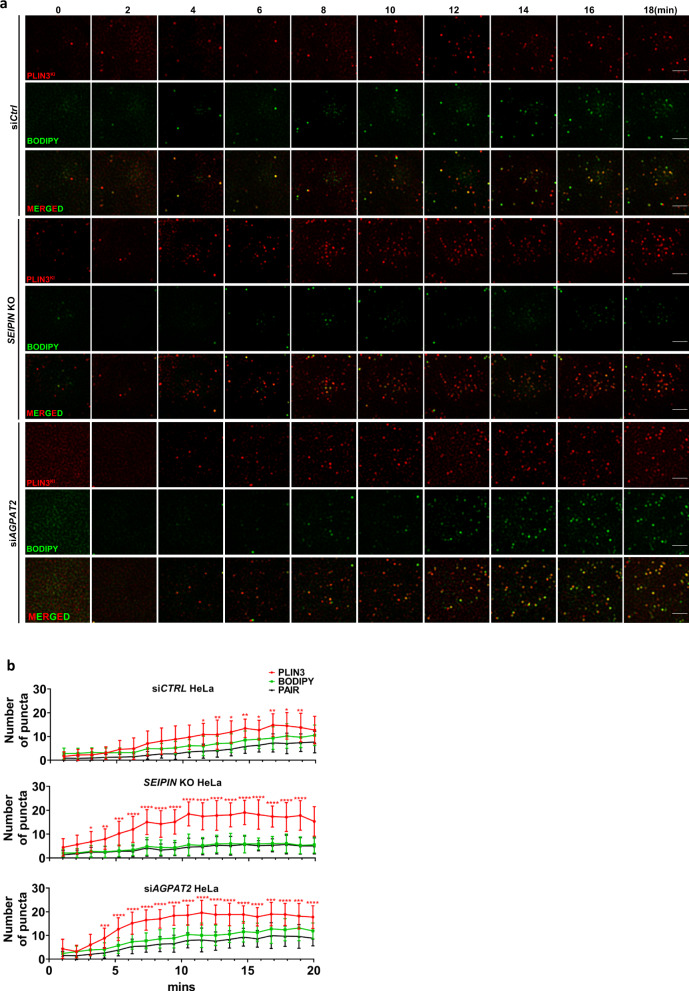
AGPAT2 depletion impacted initial LD formation. **a** Control, *seipin* knockout (KO) and *AGPAT2* knockdown HeLa cells deficient were starved in 1%LPDS for 16 h. Representative images show the localization pattern of endogenous PLIN3 (mCherry-tagged) and BODIPY puncta every two minutes after oleate addition (400 µM). Bars: 5 μm. **b** The number of PLIN3 and BODIPY puncta in control, *seipin* KO and *AGPAT2* knockdown HeLa cells at indicated time points. Pair indicates colocalization of PLIN3 and BODIPY puncta. (mean ± SD; ****p < 0.0001; ***p < 0.001; **p < 0.01; *p < 0.05, two-way ANOVA, *n* = 15–20 cells examined over three biologically independent experiments).

LDs are believed to originate from the ER via several steps. First, TAGs are synthesized within the two leaflets of the ER by diacylglycerol acyltransferases (DGATs)^26,27^. When newly synthesized TAG reaches a critical mass, it is thought to nucleate and bud from discrete sites of the ER as initial LDs. This nucleation and budding process may be controlled by both proteins such as seipin and/or ER membrane phospholipids and surface tension^22,23,28^. Given that PA, a negatively charged conical lipid, was dramatically increased in AGPAT2-deficient cells^11–13^, we wondered if AGPAT2 deficiency may also impact the early steps of LD formation. To investigate this possibility, we tagged endogenous perilipin 3 (PLIN3) by genome engineering with mCherry as described^23^. PLIN3 is an endogenous protein that indicates the earliest steps of LD formation whereas lipophilic dyes such as LipidTox/BODIPY only stain LDs that have acquired substantial neutral lipids. We also used seipin knockout cells as a control because seipin deficiency is known to impair early LD formation^23,29^. In WT cells, when LD formation was induced by oleate addition, PLIN3 rapidly accumulated in small puncta that grew larger, and became BODIPY-positive within 20 min (Fig. 2a, b). In both seipin- and AGPAT2-deficient cells, there was a sharp increase in the number of PLIN3 puncta within 10 min of oleate treatment (Fig. 2a, b; Supplementary Fig. 2a). Moreover, there were much fewer BODIPY-positive LDs in AGPAT2-deficient cells within 10 min of oleate treatment than in WT cells (Fig. 2a, b; Supplementary Fig. 2b). In seipin-deficient cells, the delay in forming BODIPY-positive LDs is more pronounced as few PLIN3 puncta became BODIPY-positive after 18 min of oleate treatment (Fig. 2a, b; Supplementary Fig. 2b).

### Reducing PA restores normal LD morphology in AGPAT2-deficient cells

**Fig. 3:**
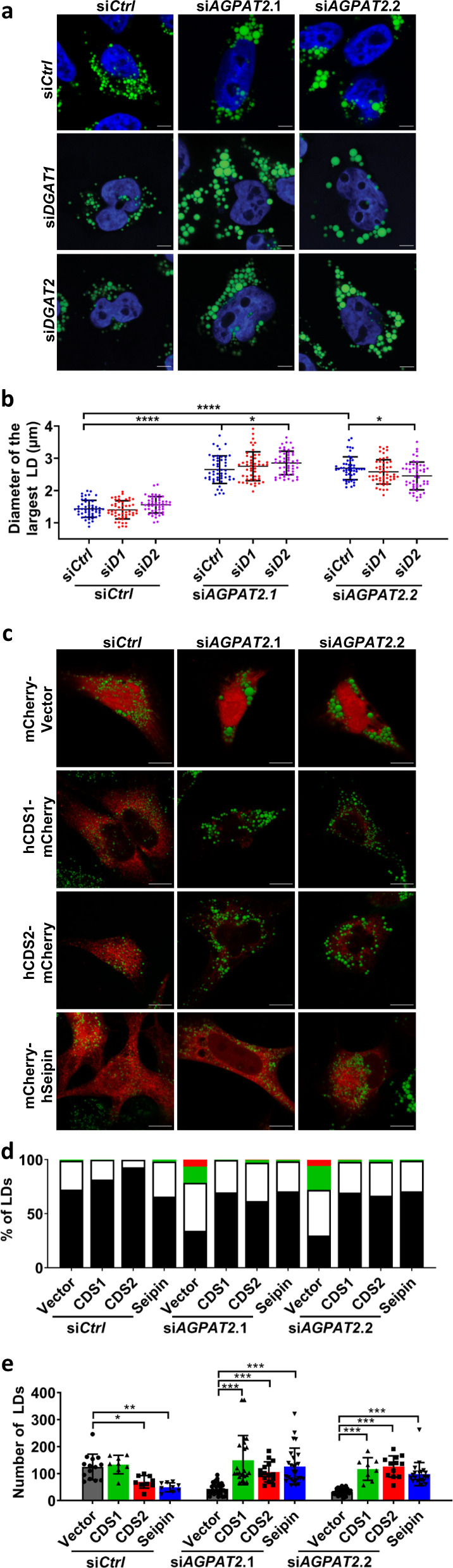
Supersized LDs in AGPAT2-depletion cells can be rescued by reducing PA. **a** LD morphology after knocking down *DGAT1 or DGAT2* in *AGPAT2*-deficient cells HeLa cells by siRNA. Cells were treated with 400 μM oleic acid for 18 h. Blue represents DAPI staining, and green represents BODIPY staining. Bars = 5 μm. **b** Quantification of LD diameters as shown in (**a**). mean ± SD; ****p < 0.0001; *p < 0.05, two-way ANOVA, *n* = 50 cells examined over three biologically independent experiments. **c** LD morphology after overexpressing hCDS1-mCherry, hCDS2-mCherry and mCherry-hSeipin in *AGPAT2* knockdown HeLa cells. Cells were treated with 400 μM oleic acid for 18 h. Green represents BODIPY staining and red represents mCherry expression. Bars = 10 μm. **d** and **e** Distribution of LDs according to diameters (**d**) and the number of LDs (**e**) after the over-expressing mCherry tagged-CDS1, CDS2 and seipin in control and AGPAT2-deficient cells. **d** LD size was represented by red (>3 µm), green (2–3 µm), white (1–2 µm) and black (0–1 µm). **e** mean ± SD; ***p *<* 0.001; **p *<* 0.01; *p *<* 0.05, two-way ANOVA, *n* = 15–30 cells examined over three biologically independent experiments.

We next aimed to determine the molecular basis for the aberrant LD biogenesis and formation under AGPAT2 deficiency. Knocking down DGAT1 or 2 with established siRNAs did not completely restore the normal size of LDs in AGPAT2-deficient cells (Fig. 3a, b)^30^. This suggests that mechanisms other than TAG synthesis may also be responsible for increased LD size. As mentioned above, an increase in whole cell PA in AGPAT2-deficient cells has been reported by different groups^11–13^. This was further confirmed here by using a PA sensor (GFP-PDE4A1) in AGPAT2 knockdown HeLa cells^31^: total fluorescence intensity of GFP-PDE4A1 in AGPAT2-deficient cells was higher than that in control cells, and GFP-PDE4A1 colocalized with calnexin, suggesting increased PA in the ER (Supplementary Fig. 3a, b). Among other possibilities, this increase in PA may underpin the formation of large LDs in AGPAT2-deficient cells. To examine this possibility, we overexpressed seipin, CDS1 and CDS2. Overexpressing any one of these three genes reduced cellular PA (Supplementary Fig. 3c, d) and almost completely abolished the formation of supersized LDs in AGPAT2-deficient cells (Fig. 3c–e). Together, these results suggest that increased PA is at least partially responsible for the abnormal LD formation in AGPAT2-deficient cells.

### Reduced CDS protein expression and activity in AGPAT2-deficient cells

**Fig. 4:**
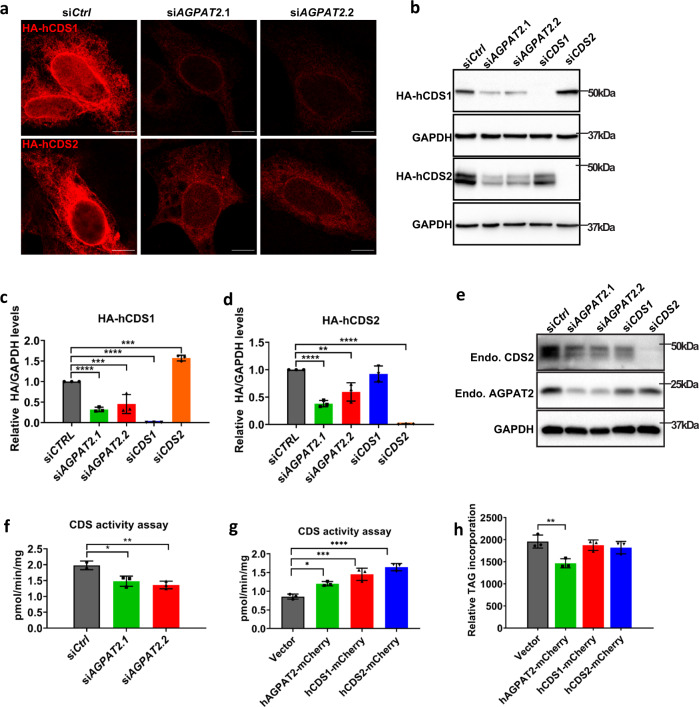
Reduced CDS1/2 protein level and activity in AGPAT2-deficient cells. **a** The fluorescence intensity of HA-hCDS1/2 in control and *AGPAT2* knockdown Huh7 cells. Bars: 10 μm. **b** Immunoblot of HA-tagged CDS1 and CDS2 in control, *AGPAT2*, *CDS1* and *CDS2* knockdown Huh7 cells. **c**, **d** Quantitation of (**b**) (mean ± SD; one-way ANOVA ****p < 0.0001, *n* = 3 biologically independent experiments). **e** Immunoblot of endogenous CDS2 and AGPAT2 protein in control, *AGPAT2* and *CDS2* knockdown Huh7 cells. **f** CDS activity in control and *AGPAT2* knockdown Huh7 cells. CDS activity assay was performed by using [^3^H] cytidine 5′-phosphate, egg PA and 0.05 mg membrane fractions from Huh7 transiently transfected with control or *AGPAT2* siRNA. The radioactive products were measured by a scintillation counter (mean ± SD; one-way ANOVA, **p < 0.01, *p < 0.05, *n* = 3 biologically independent experiments). **g** CDS activity in HeLa cells transiently transfected with mCherry-vector, hAGPAT2-mCherry, hCDS1-mCherry or hCDS2-mCherry (mean ± SD; one-way ANOVA, ***p < 0.001, **p < 0.01, *p < 0.05, *n* = 3 biologically independent experiments). **h** TAG incorporation rate was measured by treating HeLa cells transiently transfected with mCherry-vector, hAGPAT2-mCherry, hCDS1-mCherry or hCDS2-mCherry with 1 µCi [^14^C] oleate for 30 min. ^14^C-labelled TAG was extracted, separated, and visualized by Typhoon FLA 9500 phosphor imager (mean ± SD; one-way ANOVA, **p < 0.01, *n* = 5 biologically independent experiments).

While examining the effect of CDS1/CDS2 expression on LD formation in AGPAT2-deficient cells, we noticed that the fluorescence intensity of CDS1/2, but not seipin, was much weaker in AGPAT2-deficient than WT HeLa cells (red fluorescence, Fig. 3c). We further verified this observation in Huh7 cells (Fig. 4a and Supplementary Fig. 4a). The loss of mCherry-CDS1/2 signal in AGPAT2-deficient cells suggests a possible reduction of CDS1/2 protein mass and activity. This could explain the increased PA in AGPAT2-deficient cells because CDS1/CDS2-deficiency is known to result in the accumulation of PA and giant LDs^15,30,32^. We therefore examined the amount of CDS1 and CDS2 in WT and AGPAT2-deficient cells. Consistent with the imaging results, both HA-CDS1 and -CDS2 were reduced in AGPAT2-deficient cells (Fig. 4b–d). Moreover, the amount of endogenous CDS2 protein was significantly decreased in AGPAT2-deficient Huh7 cells (Fig. 4e). We were not able to quantify endogenous CDS1 due to the lack of a suitable antibody. The reduction in CDS1 and CDS2 was not due to transcriptional regulation, since CDS1/2 mRNA did not decrease in AGPAT2-deficient cells (Supplementary Fig. 4b). Similarly, AGPAT2 mRNA expression was not affected in CDS1/2 deficient cells despite a minor reduction in AGPAT2 protein (Fig. 4e and Supplementary Fig. 4b). The half-life of CDS1/2 is about 4–8 h in WT cells (Supplementary Fig. 4c–e). Under AGPAT2 deficiency, the steady state level of CDS1/2 was dramatically reduced and the effect of cycloheximide (CHX) became less clear (Supplementary Fig. 4c–e). Consistent with the reduction in protein expression, CDS activity was decreased by ~30% in *AGPAT2*-deficient cells relative to control cells (Fig. 4f). Moreover, overexpressing AGPAT2, CDS1 or CDS2 increased CDS activity (Fig. 4g and Supplementary Fig. 4f). Overexpressing AGPAT2 also caused a small but significant decrease of ^14^C oleate incorporation into TAG (Fig. 4h and Supplementary Fig. 4g). Finally, since there are five isoforms of AGPAT, we wonder if the loss of CDS1/2 was AGPAT2 specific. Knocking down AGPAT2, but none of the other AGPATs, consistently decreased CDS1/2 (Supplementary Fig. 4h–k). Together, these results support a specific functional relationship between AGPAT2 and CDS1/2.

### AGPAT2 and CDS1/2 physically interact and form stable complexes

**Fig. 5:**
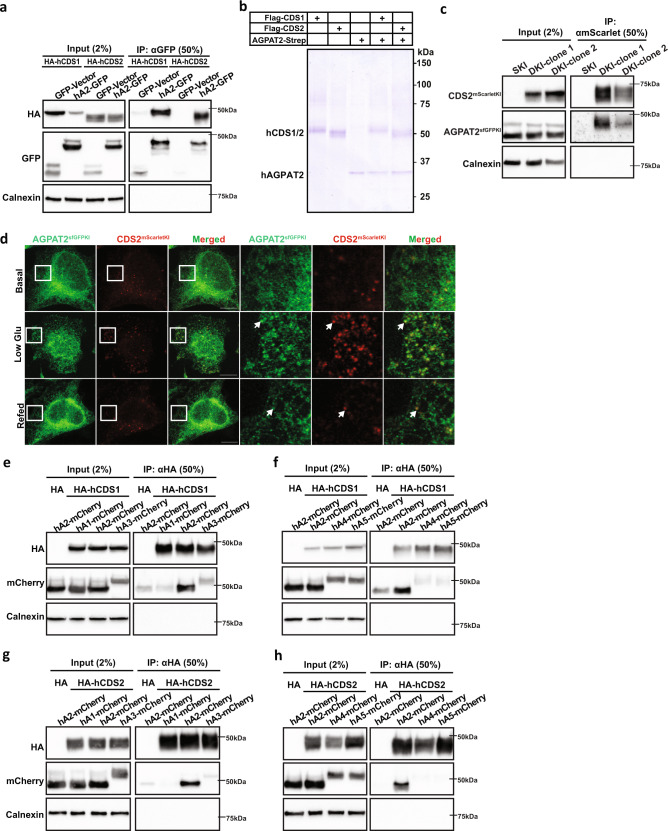
AGPAT2 and CDS1/2 physically interact. **a** Co-immunoprecipitation assay showing the interaction between AGPAT2-GFP and HA-CDS1/2 in HEK296E cells. *n* = 3 biologically independent experiments. **b** Strep-tagged AGPAT2 and Flag-tagged CDS1 or CDS2 were expressed in HEK293F cells alone or together as indicated, followed by either a one-step affinity purification by anti-Flag or anti-Strep or a two-step affinity purification by anti-Flag and then anti-Strep. The Coomassie blue-stained gel shows purification of CDS1, CDS2 and AGPAT2, and co-purification of CDS1/2 and AGPAT2. *n* = 3 biologically independent experiments. **c** Co-immunoprecipitation assay showing the interaction between endogenous AGPAT2-sfGFP and CDS2-mScarlet in HeLa cells. AGPAT2 or CDS2 was tagged at their genomic locus with sfGFP or mScarlet by CRISPR. SKI: single sfGFP knockin at AGPAT2. DKI: mScarlet knockin at CDS2 and sfGFP knockin at AGPAT2. *n* = 3 biologically independent experiments. **d** Confocal imaging of fixed HeLa cells showing AGPAT2 and CDS2 tagged at their genomic loci with sfGFP and mScarlet, respectively. Cells were treated with high glucose DMEM (basal) or low glucose DMEM for 48 h or re-incubation with high glucose DMEM (Refed) for another 24 h. Bars = 10 μm. *n* = 3 biologically independent experiments. **e** Co-immunoprecipitation of mCherry-tagged AGPAT1, 2, 3 and HA-tagged CDS1 from transfected HEK293E lysates. *n* = 3 biologically independent experiments. **f** Co-immunoprecipitation of mCherry-tagged AGPAT2, 4, 5 and HA-tagged CDS1 from transfected HEK293E lysates. *n* = 3 biologically independent experiments. **g** Co-immunoprecipitation of mCherry-tagged AGPAT1, 2, 3 and HA-tagged CDS2 from transfected HEK293E lysates. *n* = 3 biologically independent experiments. **h** Co-immunoprecipitation of mCherry-tagged AGPAT2, 4, 5 and HA-tagged CDS2 from transfected HEK293E lysates. *n* = 3 biologically independent experiments.

One possible explanation for the loss of CDS1/2 under AGPAT2 deficiency is that AGPAT2 may form a complex with CDS1/2 and promote their stability/activity. The interaction between AGPAT2 and CDS1/2 was therefore investigated. Human CDS1 and CDS2 both co-immunoprecipitated with human AGPAT2 with similar affinity (Fig. 5a, Supplementary Fig. 5a). As a control, seipin interacted with GPAT4, but not with AGPAT2 under the same conditions (Supplementary Fig. 5b). To further examine the AGPAT2-CDS interaction, we also expressed Strep-tagged AGPAT2 and Flag-tagged CDS1 or CDS2 in HEK293F cells, followed by a two-step affinity purification by anti-Flag and anti-Strep. A large amount of AGPAT2 and CDS1/2 co-purified as assessed by Coomassie blue, suggesting a direct and stable interaction (Fig. 5b and Supplementary Fig. 5c). To verify the interaction between AGPAT2 and CDSs, we tagged AGPAT2 with sfGFP and CDS2 with mScarlet at their respective genomic locus by CRISPR (Supplementary Fig. 5d). For unknown reasons, we were not able to tag CDS1 despite multiple attempts. Notably, knocking down AGPAT2 not only reduced the level of AGPAT2–sfGFP, but also that of CDS2-mScarlet, further demonstrating that AGPAT2 is required for the stability of CDS2 as shown in Fig. 4 (Supplementary Fig. 5d). Importantly, AGPAT2–sfGFP and CDS2-mScarlet coprecipitated (Fig. 5c). There are two isoforms of AGPAT2 and CDS2 appears to interact stronger with the longer isoform (Fig. 5c and Supplementary Fig. 5d). As a further proof of the dynamic interaction between AGPAT2 and CDS2, there appears to be enhanced colocalization between endogenous AGPAT2 and CDS2 when cells were cultured in low glucose media (Fig. 5d and Supplementary Fig. 5e). The enhanced colocalization was reversed upon adding back glucose. We further determined if the apparent interaction between AGPAT2 and CDS1/2 is unique since there are five AGPAT isoforms. Co-immunoprecipitation (co-IP) experiments using AGPAT1–5 and CDS1/2 identified AGPAT2 as the major AGPAT isoform to co-precipitate with CDS1/CDS2 (Fig. 5e–h; Supplementary Fig. 5f–i), consistent with its specific functional link with CDS1/2.

### AGPAT2 promotes the flux of oleate through the CDP-DAG pathway

**Fig. 6:**
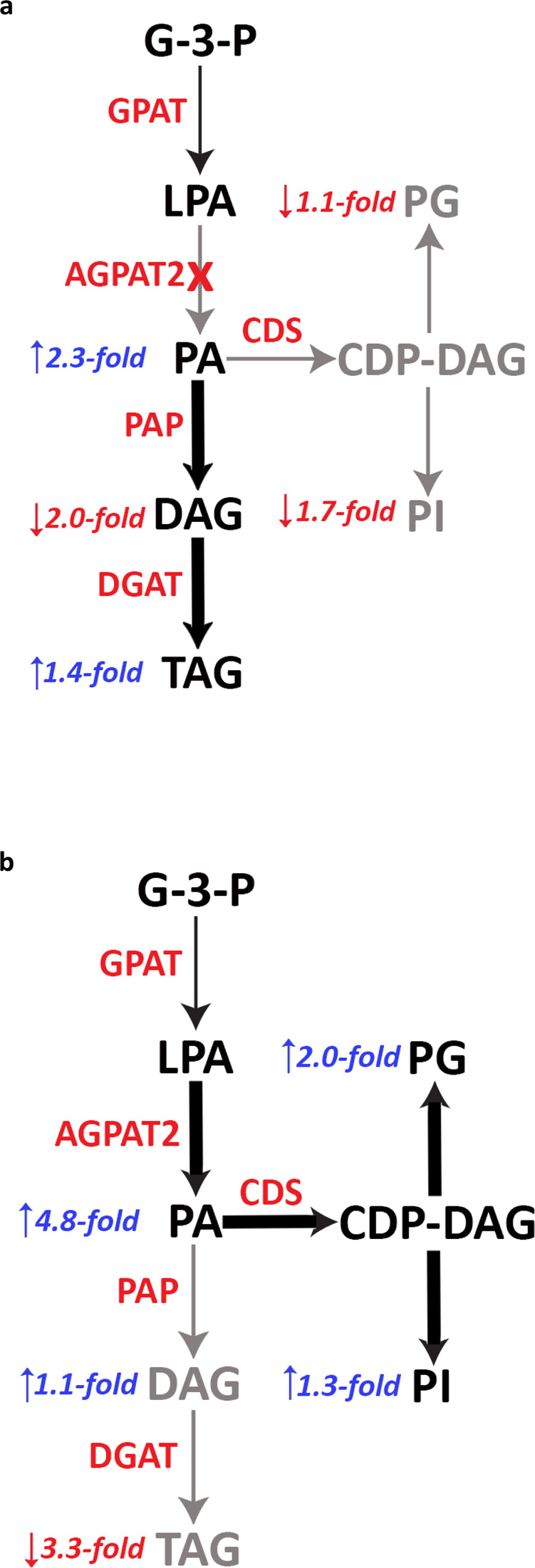
AGPAT2 promotes oleate incorporation into phospholipids of the CDP-DAG branch. HeLa cells were transfected with AGPAT2 siRNA (**a**) or human AGPAT2-mCherry (**b**) for 24 h. Cells were then treated with dialysed FBS for 16 h and then for 8 h with 10 µM [U^13^C]-oleate (C18:1). ^13^C-labelled samples were analysed by LC–MS. Fold changes of oleate incorporation were indicated as blue (increase) and red (decrease) arrows.

PA generated by AGPATs is a key branch point metabolite in the synthesis of phospholipids and TAGs: PA can be used directly by CDS1/CDS2 for the synthesis of CDP-DAG or by PAPs (e.g., lipins) for the synthesis of DAG. Substrate channelling often occurs at metabolic branch points^33,34^. AGPAT2 and CDS1/2 may form specific stable complexes to facilitate the delivery of PA to CDS1/2. To test this hypothesis, we carried out metabolic flux analyses using ^13^C-oleate as a tracer. We first conducted a time course experiment to assess ^13^C-oleate incorporation into phospholipids and TAGs (Supplementary Fig. 6a, b). We then conducted further analyses at the 8 h time point. Knocking down AGPAT2 reduced oleate incorporation into PI by 1.7-fold and to a lesser extent, into PG, while incorporation into TAG increased by ~40% (Fig. 6a). Conversely, overexpressing AGPAT2 increased oleate incorporation into PG (by ~100%) and PI (by ~30%) and reduced the flux to TAG by 3.3-fold (Fig. 6b). As a control, overexpressing AGPAT1 had a modest effect on the flux to PG and PI (Supplementary Fig. 6c). The rate of ^13^C-oleate incorporation into different lipids was shown in Supplementary Fig. 6d, e.

### AGPAT2-deficient liver has reduced CDS2 protein level and CDS activity in vivo

**Fig. 7:**
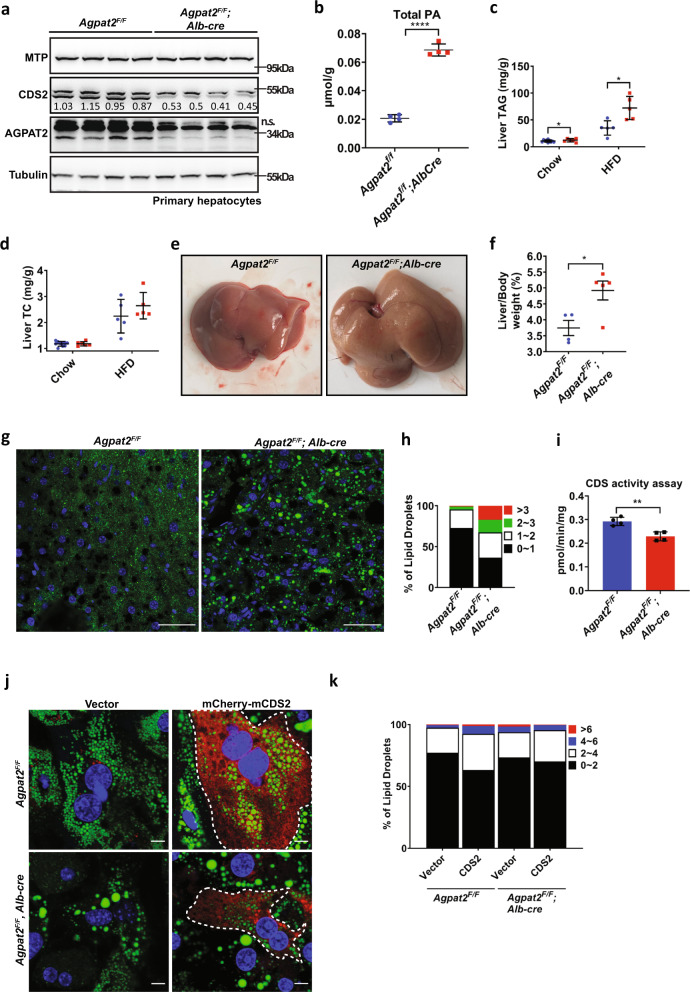
Ablation of AGPAT2 from mouse liver reduces hepatic CDS activity. **a** Western blotting of MTP, CDS2 and AGPAT2 from primary hepatocytes isolated from WT (*Agpat2*^*f/f*^, *n* = 4) and liver-specific AGPAT2 knockout mice (A2LKO/*Agpat2*^*f/f*^*, Alb-cre*, *n* = 4). **b** Total PA levels of WT (*n* = 4) and A2LKO (*n* = 4) mice fed chow diet. **c** Liver TAG and **d** total cholesterol (TC) of WT (*n* = 6–9) and A2LKO (*n* = 5–7) mice fed chow or HFD. **e** Liver morphology and **f** the ratio of liver to body weight from WT and A2LKO mice fed HFD. **g** BODIPY-stained liver sections from WT and A2LKO mice fed HFD. Bars: 50 μm. **h** Distribution of LDs of different diameters as shown in (**g**). **i** CDS activity of WT (*n* = 4) and A2LKO liver (*n* = 4). **j** Primary hepatocytes from WT and A2LKO chow-fed mice were transfected with mCherry-CDS2 and stained with BODIPY. Dashed line indicates cells transfected with mCherry-CDS2. **k** Distribution of LDs according to diameters as shown in (**j**). **b**–**d**, **f** and **i** mean ± SD; two-tailed unpaired *t* test. *p < 0.05, **p < 0.01, ****p < 0.0001.

To confirm our findings on the specific functional connection between AGPAT2 and CDS1/2 in vivo, we generated a liver-specific AGPAT2 knockout mouse (A2LKO mice) by CRISPR/Cas9-mediated gene editing. The strategy used to disrupt *Agpat2* in mice is shown in Supplementary Fig. 7a: two Lox*P* sites were introduced into the *Agpat2* locus, sandwiching exons 2 and 4. Homozygous *Agpat2*^fl/fl^ mice were then crossed with transgenic mice expressing *Cre* recombinase under the control of the albumin promoter. The resulting Agpat2^fl/+^Alb^−*cre*Tg/0^ progeny was then crossed with *Agpat2*^fl/fl^ mice to generate the A2LKO mice. Littermates lacking the *Cre* gene (*Agpat2*^fl/fl^) were used as controls and referred to as WT. The successful genomic disruption of *Agpat2* was confirmed by the absence of the *Agpat2* mRNA transcript and protein using western blotting and real-time quantitative PCR (Fig. 7a; Supplementary Fig. 7b). The level of PA in A2LKO liver increased almost three-fold (Fig. 7b), consistent with previous reports using other cell types/tissues^11–13^. Distinct from the global *Agpat2*-deficient (*Agpat2*^*−/−*^) mice, the A2LKO mice have normal brown and white adipose tissue weight (BAT and WAT) (Supplementary Fig. 7c, d). Food intake, body weight, liver and gastrointestinal weight of the A2LKO mice also appear normal (Supplementary Fig. 7e–h). Expression analyses showed little change in other AGPATs, CDS1/2 and most key metabolic genes in the liver of A2LKO mice except a mild (50%) increase in ACC2 (Supplementary Fig. 7i).

There was an increase of TAG but not total cholesterol (TC) in the liver of A2LKO mice fed either chow or high fat diet (HFD) (Fig. 7c, d). There also appeared to be slightly more and larger LDs in A2LKO liver on chow diet (Supplementary Fig. 7j, k). Notably, the liver of A2LKO mice on HFD appeared pale and enlarged, and the size of LDs increased dramatically, consistent with data from cell lines (Fig. 7e–h). Most importantly, the levels of hepatic CDS2 protein and hepatic CDS activity were significantly reduced in A2LKO mice (Fig. 7a and i). Moreover, oleate treatment of primary hepatocytes isolated from A2LKO mice resulted in enlarged LDs, which was reversed by overexpressing mCherry-CDS2 (cells with red signal) (Fig. 7j, k). Overall, these results are consistent with data from cell lines and pull-down assays, and further support the functional relationship between AGPAT2 and CDS1/2.

## Discussion

The mammalian genome encodes five putative AGPAT isoforms that catalyze a key step in the synthesis of phospholipids and TAGs: the acylation of LPA to PA. AGPAT2 is the only AGPAT isoform whose loss-of-function mutations are associated with *BSCL1/CGL1*^9^. It has been puzzling that the direct product of AGPAT2, PA, is increased in AGPAT2-deficient cells^11–13^. Our results from cellular studies, liver specific AGPAT2 knockout mice as well as biochemical analyses reveal that: 1. AGPAT2 regulates the biogenesis of cytoplasmic LDs; 2. The stability and activity of CDS enzymes are decreased in AGPAT2-deficient cells and mouse liver, contributing to the increased PA under AGPAT2 deficiency; 3. AGPAT2 and CDS1/2 form a stable functional complex possibly to channel PA for the synthesis of CDP-DAG and phospholipids of the CDP-DAG branch, e.g., PI and PG. Most importantly, our results imply substrate channeling at a major branch point of the glycerol-3-phosphate pathway.

The immediate product of AGPAT2, PA, sits at a metabolic branch point for the synthesis of all phospholipids and TAGs (Fig. 1a). PA can be de-phosphorylated into DAG by PAPs (i.e. the lipins) for the synthesis of PC, PE, PS and TAG^35,36^; PA can also be converted into CDP-DAG through the action of CDS1/2 for the synthesis of PI, and possibly PG and CL^8,32^. Little is known about how the flux through this important metabolic branch point is regulated. Substrate channeling plays a key role in controlling flux at network branch points: the channeled metabolic intermediate is transferred directly from one enzyme to the next of the same biosynthetic pathway and is therefore prevented from being used by competing branch-point reactions^33,34^. The channeling is often facilitated by the formation of specific enzyme assemblies/protein complexes. An AGPAT2–CDS1/2 complex would channel PA towards the synthesis of CDP-DAG, thereby decreasing its reaction with PAPs for DAG synthesis. Indeed, AGPAT2 is the only AGPAT isoform that specifically and directly interacts with CDS1 and CDS2. Strikingly, CDS proteins became unstable and CDS activity was significantly reduced under AGPAT2 deficiency, in both cell lines and mouse liver. Moreover, overexpressing AGPAT2 increased CDS activity, and reduced oleate incorporation into TAG. Consistently, metabolic flux analyses showed that the rate of oleate incorporation into PG/PI (the CDS branch) was increased upon overexpressing and decreased upon knocking down AGPAT2. It is also worth noting that the steady-state concentrations of PI and PG were significantly reduced in AGPAT2-deficient mouse embryonic fibroblasts^12^, consistent with our present observations. Together, our data provide physical and functional evidence that AGPAT2 and CDS1/2 form complexes to promote the flux of PA into the CDP-DAG pathway. Without AGPAT2, CDS activity is reduced, causing accumulation of PA, aberrant LD formation and impaired adipogenesis^30,32^. Although CDS1/2 and AGPAT2 can form rather stable complexes which can be co-purified in large quantity, we were unable to resolve the structures of the two complexes at this time despite multiple attempts. While CDS1 and CDS2 can both interact with AGPAT2, we were unable to detect a stable interaction between CDS1 and CDS2 (data not shown). Thus, AGPAT2 may associate with CDS1 or CDS2 separately, and the resulting distinct complexes may operate at different regions of the ER, and/or catalyze the synthesis of different CDP-DAG species, and/or function under different conditions. Future efforts are required to obtain insights into the structural details of the AGPAT2–CDS1/2 complexes and their specific functions.

The finding that AGPAT2’s product, PA, is increased in AGPAT2-deficient cells and tissues has been confusing and paradoxical. We show here that the reduced CDS activity under AGPAT2 deficiency may be at least partially responsible for the increase in PA. Indeed, overexpressing CDS1/2 reduced the accumulation of PA and restore normal LD formation in AGPAT2-deficient cells. Although CDS1/2 can clearly form functional complexes with AGPAT2, our results also suggest that CDS1/2 can use PA generated from other sources (e.g. other AGPATs, DAG kinases or phospholipase D)^37^. Under AGPAT2 deficiency, CDS activity and the flux of oleate to PG and PI were significantly but only moderately reduced, suggesting that PA from other sources is available for use by the remaining CDS enzymes. There also appears to be increased flux of oleate to PA synthesis in AGPAT2-deficient cells. Therefore, AGPAT2 deficiency may trigger a range of biochemical changes in addition to impaired CDS stability and activity, and the molecular basis for those changes requires further investigation.

Our results also reveal cell autonomous roles of AGPAT2 in LD formation. AGPAT2 deficiency delayed the maturation of initial LDs and formed supersized LDs after prolonged oleate treatment, phenotypes reminiscent of seipin deficiency^14,17,24,29,38^. The delay in early LD lipidation in AGPAT2-deficient cell is not as severe as that in seipin knockout cells. It should be noted that while seipin was knocked out, AGPAT2 was knocked down, because the AGPAT2 knock-out cancer cell lines were very sick in our hands and therefore not used. Nevertheless, these results further connect *BSCL1* (AGPAT2) with *BSCL2* (seipin) beyond adipogenesis. As PA has been implicated in seipin function^15–18,39^, the increased PA in the ER may also underpin the effect of AGPAT2 deficiency on LD dynamics. Indeed, aberrant LD formation under AGPAT2 deficiency can be rescued by overexpressing CDS1/2 (consuming PA) or seipin (reducing PA production and increasing PA sequestration)^16,17^. Thus, our results highlight the role of non-bilayer lipids (e.g. PA) in LD biogenesis possibly by modulating the surface tension and curvature at sites of LD formation^22^. To investigate the function of AGPAT2 in vivo, we generated the liver specific A2LKO. The LD phenotypes in AGPAT2-deficient liver and primary hepatocytes are consistent with those observed in cell lines. Importantly, CDS activity was significantly reduced in A2LKO liver, and the LD phenotype in AGPAT2-deficient primary hepatocytes was rescued by overexpressing CDS2. Overall, these results suggest that the reduced CDS activity in AGPAT2-deficient hepatocytes led to accumulation of PA in the ER, forming enlarged cytoplasmic LDs.

Although our data from biochemical assays, cell line and mouse studies strongly support a role for AGPAT2 to promote the synthesis of CDP-DAG branch of phospholipids, there are some limitations. We could not directly visualize the channeling of PA towards CDP-DAG synthesis in the AGPAT2–CDS1/2 complex. This may be achievable after the structures of AGPAT2–CDS1/2 complexes are resolved. There is also some limitation in our flux studies because ^13^C-oleate can be incorporated into pre-existing phospholipids by the deacylation–reacylation pathway. However, it should be noted that this pathway may be a minor contributor to ^13^C-oleate incorporation into phospholipids compared with the de novo synthesis pathway.

In summary, we provide strong evidence that AGPAT2 and CDS1/2 can form stable complexes which promote CDP-DAG synthesis. We also demonstrate a role for AGPAT2 in normal LD formation. Together, these results provide key insights into the regulation of the glycerol-3-phosphate pathway, unveil the molecular basis for the increase in PA under AGPAT2 deficiency and for the pathogenesis of *BSCL1*, and open future avenues of investigation on how the metabolism of PA is controlled at a major metabolic branch point.

## Methods

### Antibodies, chemicals, plasmids, and primers

Antibodies, chemicals and plasmids with source and catalogue numbers are described in Supplementary Table 1. Primers used for cloning, for PCR and for knock-in (KI) are listed in Supplementary Tables 2–4.

### Cell culture, RNAi, and transfection

AML12, HeLa and Huh7 cells were grown in Dulbecco’s modified Eagle medium (DMEM) with 10% foetal bovine serum (FBS) and 1% penicillin–streptomycin–glutamine (PSG). Cells were maintained in 37 °C incubator with 5% CO_2_. Medium was changed every 2 days. AML12 cells were grown in DMEM:nutrient mixture F-12 (DMEM/F12) with 10% FBS, 1% insulin–transferrin–selenium (ITS-G) and 40 ng/mL dexamethasone. 3T3-L1 cells were grown in DMEM with 10% Newborn calf serum (NCS) and 1% PSG.

Transient plasmid transfection was carried out by using Lipofectamine LTX. Plasmid DNA and Lipofectamine LTX were diluted in Opti-MEM™ I Reduced Serum Media separately, followed by 5 min incubation in room temperature. The mixture was incubated for another 10 min after mixed and added to the cells culture. Cells were harvested 48 h post transfection.

Transient small interfering RNA (siRNA) transfection was carried out by using Lipofectamine RNAiMAX. 20 μM siRNA and RNAiMAX (twice the volume of siRNA) were diluted in Opti-MEM™ I Reduced Serum Media separately, followed by 5 min incubation at room temperature. The mixture was incubated for another 20 min and added to cell culture. Cells were harvested 48 h post transfection.

### Generation of KI cells with CRISPR/Cas12a-mediated genome editing

C-terminally mCherryTag-tagged PLIN3 was generated by CRISPR/Cas9 gene editing^40^. HeLa cells were simultaneously transfected by Lipofectamine™ LTX Reagent with PLUS™ Reagent (#15338100, Thermo Fisher Scientific) with Megamer^®^ Single-Stranded DNA Fragments (Integrated DNA technologies) containing arms with 100-nucleotide-long homology upstream and downstream of the target site and gRNA targeting upstream of stop codon. The single-stranded DNA fragments and gRNA information are described in Supplementary Table 3. C-terminally superfolderGFP-tagged AGPAT2 and mScarlet-tagged CDS2 was generated by CRISPR/Cas12a gene editing method^41^. A PCR cassette, containing sfGFP/mScarlet, a Cas12a CRISPR RNA and ~100 bp homology arms, were amplified from pMaCTag-P06 by Phusion™ high-fidelity DNA polymerase (#F-530L, Thermo Fisher Scientific). Primers were designed by http://www.pcr-tagging.com/. HeLa cells were simultaneously transfected by Lipofectamine™ LTX Reagent with PLUS™ Reagent (#15338100, Thermo Fisher Scientific) with equal amount of pcDNA3.1-hLbCpf1(TYCV) and PCR cassettes.

For PLIN3-mCherry KI, CDS2-mScarlet KI and AGPAT2-sfGFP KI, cells were then selected with 2 μg/mL puromycin for 48 h and recovered for 1–2 weeks in the absence of puromycin. Single-cell FACS sorting was performed by BD FACSMelody™ Cell Sorter (BD Biosciences) at the flow cytometry (UNSW). 561 and 488 nm laser were used to sort mCherry and sfGFP-positive cells, respectively. To validate the insertion of tags, target regions were amplified by PCR and sequenced.

### Viral stable transduction

LentiX-293T cells were used for lentiviral stable knockdown. LentiX-293T cells were plated at 2 × 10^6^ cells per 10 cm dish 24 h prior to transfection. Cells were transfected by using Lipofectamine LTX. For lentiviral production, 10 μg AGPAT2 shRNA, 2.970 μg pMD.G, 5.294 μg pMDLg/pRRE and 1.848 μg pRSVrev were transfected to LentiX-293T cells, followed by incubation in 37 °C with 5% CO_2_ for 48 h. Media with virus were collected from LentiX-293T cells and filtered through a 0.45 μM filter, followed by the addition of 8 μg/mL polybrene. Lentivirus titre was tested by Lenti-X GoStix Plus. The filtered viral media was then added to the 3T3-L1 adipocytes and incubated for 24 h.

### Adipocyte differentiation

To induce adipocyte differentiation, 3T3-L1 preadipocytes were grown in DMEM with 10% NCS and 1% PSG until 10% confluency. Two days post-confluency, differentiation was stimulated by using DMEM containing 10% FBS, 1% PSB and supplemented with insulin (10 µg/mL), dexamethasone (1 µM) and isobutylmethyxantine (IBMX) (0.5 mM). An additional 2 days later, cells were grown in DMEM/FBS/PSG with insulin (10 µg/mL). Media (DMEM/FBS/PSG) was refreshed every 2 days until the end of the differentiation.

### Cell proliferation assay

3T3-L1 cells were grown and differentiated in a 96-well plate. Cells were transduced with control and *AGPAT2* shRNA lentivirus at day 6 of differentiation. Cell proliferation Assay was performed at day 8 of differentiation by using CellTiter 96^®^ AQueous One Solution (Promega) according to manufacturer’s protocol.

### LDs studies

For fixed samples, cells were treated with 400 µM oleate-coupled BSA in DMEM/FBS/PSG for 18 h. Cells were rinsed twice with PBS and postfixed with 4% paraformaldehyde for 15 min at room temperature. Cells were rinsed three times with PBS before and after staining with freshly prepared 1 µg/mL BODIPY 493/503 (Thermofisher scientific) for 15 min. For immunofluorescence staining, cells were subsequently permeabilized with 0.2% TritonX-100 in PBS for 15 min at RT after fixation, and then blocked by incubation with 3% (w/v) BSA in PBS for 1 h at RT. Cells were than incubated with primary antibody and secondary antibody in 3% (w/v) BSA for 1 h at RT, with three 5 min PBS washes in between. Coverslips were then mounted onto slides by using ProLong™ Gold Antifade Mountant with DAPI (Thermofisher Scientific). All images were obtained by ZEISS LSM 900 with Airyscan microscopy (Carl Zeiss, Jena, Germany) with ×63 Plan Apochromat (1.4 NA) oil objective. Acquired images were quantified by ImageJ software.

### Live cell imaging

For live-cell imaging, HeLa cells were cultured in in DMEM/10%FBS/PSG. Pre-LDs were removed by starving the cells with 1% LPDS in DMEM for 16 h. All live cell imaging experiments were performed at +37 °C, 5% CO_2_ in FluroBrite DMEM supplemented with 1% LPDS and ProLong Live Antifade (Thermofisher Scientific). Equal volumes of media containing two times concentrated oleate-coupled BSA (800 µM) and BODIPY (1:5000) was added to the dish immediately before image acquisition. HILO microcopy was performed using Zeiss Elyra inverted microscope equipped with a high-sensitivity Andor iXon 897 EMCCD cameras fitted with a ×63, NA 1.4 Plan-Apochromat lens. Images were obtained every 1.26 s. Acquired live cells images were deconvoluted and quantified by custom MATLAB scripts.

### SDS–PAGE and immuno-blotting

Cells were washed twice with ice-cold PBS and lysed by adding 0.1% SDS lysis buffer (1% Triton X-100, 0.1% SDS, 10 mM Tris pH 7.4, 100 mM NaCl, 1 mM EDTA, 10% glycerol and complete protease Inhibitor Cocktail tablet, EDTA-Free). Cell lysates were then incubated on ice for 15 min, followed by centrifuging at 18,000 × *g* for 10 min at 4 °C. Supernatant was then transferred to a new tube. Protein concentration was determined by using BCA protein assay kit. 40 µg protein lysate was mixed with 2× Laemmli sample buffer and then loaded to 10% SDS–PAGE gel. Electrophoresis was run at 150 V for 1 h. Proteins on the gel were then transferred to nitrocellulose membrane at 100 V for 1 h. Membranes were blocked with 5% (w/v) skim milk in TBST in room temperature for 1 h, followed by incubating with primary antibody with appropriate dilution at 4 °C for 16 h. After primary antibody incubation, membranes were wash with TBST three times for 5 min each and then incubated with secondary antibody at 1:5000 dilution in TBST for 1 h at room temperature. Membranes were washed again with TBST three times for 5 min each and developed by using enhanced chemiluminescence and BioRadChemiDoc XRS+ imager.

### Co-immunoprecipitation

Protein lysates (1 mg) were mixed with 25 µL HA-agarose beads to a final volume of 500 μL per sample by using co-IP lysis buffer (25 mM HEPES pH 7.4, 150 mM NaCl, 1 mM EDTA, 10% glycerol, 1% DDM) to normalize. Protein-beads mixture was then gently rotated at 4 °C for 16 h. Protein-beads mixture was washed three times with 150 mM wash buffer (25 mM HEPES pH 7.4, 150 mM NaCl, 0.1% NP-40). Proteins were eluted by adding 30 μl 2× Protein loading buffer without β-mercaptoethanol, followed by vortexing, and centrifuging at 18,000 × *g* for 5 min. Supernatant was then transferred to a new tube and 30 μl 2× protein loading buffer (with β-mercaptoethanol) was added. Protein lysates were then subjected to SDS–PAGE and Western blot.

### RNA extraction and quantitative real-time PCR

Total RNA was extracted using TRIzol^TM^ reagent. Mammalian cells were grown in 6 cm Petri dishes. Cells were washed with ice cold PBS once, followed by adding 1 mL TRIzol^TM^ reagent and rocking for 5 min. 200 µL chloroform was added and the mixture was shaken vigorously. After incubating for 3 min in room temperature, the mixture was then centrifuged at 12,000 × *g* for 15 min at 4 °C. After centrifugation, the top aqueous layer was transferred to a new tube and 0.5 mL 100% isopropanol was added. After 10 min incubation in room temperature, the mixture was then centrifuged again at 12,000 × *g* for 10 min at 4 °C. After centrifugation, supernatant was removed and 1 mL 75% ethanol was added, followed by vortexing for 15 s. The sample was then subjected to centrifugation at 7500 × *g* for 5 min at 4 °C. This step was repeated twice. The RNA pellet was dried at 55 °C for 10 min and dissolved in RNAse free water. cDNA synthesis was then performed by using 1 µg RNA and high-capacity cDNA reverse transcription kit. cDNA was synthesized by one cycle of denaturation at 25 °C for 10 min, annealing and extension at 37 °C for 120 min and enzyme deactivation at 75 °C for 5 min.

All qRT-PCR primers used in this project are listed in Supplementary Table 3. qRT-PCR was performed by using the KAPA SYBR FAST qRT-PCR kit. qRT-PCR samples were held at 95 °C for 4 min then followed by 40 cycles of denaturation at 95 °C for 10 s, annealing at 60 °C for 20 s and extension at 72 °C for 20 s. The mRNA expression levels were obtained by normalizing against house-keeping gene and comparing to control.

### CDS1/2**–**AGPAT2 co-purification assay

The full-length cDNA of human CDS1, CDS2 and AGPAT2 were subcloned into the pCAG vector, tagged with an N-terminal Flag tag for CDS1/2 and a C-terminal Strep tag for AGPAT2. The recombinant Flag-CDS1/CDS2 and AGPAT2-Strep were co-expressed in HEK293F cells. The cells were transiently transfected at a density of 2.0 × 10^6^ cells per mL, using polyethyleneimine (PEI) (Polysciences). A 100-mL cell culture was transfected with 0.075 mg of CDS1 or CDS2 plasmid, and 0.075 mg of AGPAT2 plasmid. After 12 h, the cell culture was supplemented with 10 mM sodium butyrate, to boost protein expression. After another 48 h, the cells were collected by centrifugation and were resuspended in buffer containing 25 mM Tris pH 8.0, 150 mM NaCl, and protease inhibitor cocktails (Amresco). Membranes were solubilized at 4 °C for 2 h with 1% (w/v) GDN (Anatrace). After centrifugation at 20,000 × *g* for 45 min, the supernatant was applied to anti-Flag G1 affinity resin (GenScript). The resin was rinsed with buffer W (25 mM Tris pH 8.0, 150 mM NaCl, 0.02% (w/v) GDN, 5 mM ATP, 5 mM MgCl_2_), and bound protein was eluted with buffer E1 (25 mM Tris pH 8.0, 150 mM NaCl, 0.02% (w/v) GDN, 200 μg/mL Flag peptide). The eluent was then applied to Strep-Tactin resin (IBA Lifesciences). After rinsing with buffer W, bound protein was eluted with buffer E2 (25 mM Tris pH 8.0, 150 mM NaCl, 0.02% (w/v) GDN, 50 mM Biotin). The eluent was analysed by SDS–PAGE and stained with Coomassie blue dyes.

### CDS activity assay

CDS activity assay was performed as described^42^. Huh7 cells were resuspended in ice cold lysis buffer (50 mM Tris–HCl (pH 8.0), 50 mM KCl, 0.2 mM ethylene glycol tetraacetic acid (EGTA), and 1/100 (v/v) protease inhibitor cocktail) and lysed by 30 passes through homogenizer. Nuclei and unbroken cells were removed by centrifugation at 1000 × *g* for 10 min at 4 °C. Membrane fraction was then extracted by removing the supernatant and centrifuging at 100,000 × *g* for 1 h at 4 °C.

200 μM Egg PA was dried down by speediVac and resuspended in 125 mM Tris–HCl (pH 8), 250 mM KCl, 12.75 mM Triton X-100, 5 mg/mL BSA and 0.625 mM DTT. CDS activity assay was performed in a 100 μl reaction. 2 μl MgCl_2_ (1 M stock) was added to 40 μl egg PA suspension, followed by 48 μl of protein sample (50 μg). The reaction was then initiated by adding 7.5 μl of CTP (200 μM stock) pre-mixed with 2.5 μCi CTP [5′-3H] and incubated for 10 min at 30 °C. This was then terminated by adding 375 μl chloroform:methanol (1:2), followed by adding 125 μl chloroform and 125 μl water. The mixture was vortexed and centrifuged in 1000 × *g* for 5 min. The lower organic phase was recovered and washed with the synthetic top phase, 125 μl methanol and water (1:0.9). The lower organic phase was evaporated in a fume hood prior to counting the radioactivity in a scintillation counter.

### ^14^C Oleate incorporation assay

2.5 × 10^5^ HeLa cells were seeded in 6-cm dishes, followed by transfection with mCherry-Vector, hAGPAT2-mCherry, hCDS1-mCherry or hCDS2-mCherry. Cells were then treated with 1 µCi [^14^C] oleic acid (PerkinElmer) conjugated to BSA in DMEM–10%FBS medium for 30 min after reaching ~80% confluency. After washing cells twice with PBS, ^14^C-labelled TAG was extracted by a 2-mL mixture of hexane (Ajax FineChem) and isopropanol (Ajax FineChem) (3:2) for 30 min in a fume hood. After transferring into 2-mL glass vials, another 1 mL of hexane:isopropanol (3:2) was used to collect the lipid residues and combined with the previous 2 mL solvent. Solvent was dried completely in the fume hood. Lipids were reconstituted in 60 μl of hexane. The samples were then spotted on a Silica Gel 60 plate (Millipore) and separated in a solvent system consisting of heptane/diethyl ether/glacial acetic acid (90:30:1) (Ajax FineChem). The TLC plate, after drying completely, was exposed to a BAS-MS imaging sheet (Fujifilm, Tokyo, Japan) for 5–7 days in an enclosed cassette at room temperature. ^14^C-labelled TAG was then visualized by Typhoon FLA 9500 phosphor imager (GE Healthcare). After the dishes were dried completely, 1 mL 0.1 M NaOH (Ajax FineChem) was added to extracted protein. The protein concentrations were determined by a bicinchoninic acid (Sigma) assay.

### ^13^C-FA tracing and lipid extraction

2 × 10^5^ HeLa cells were seeded overnight in six-well plates, followed by transfection with siRNA against *AGPAT2* or mCherry-tagged AGPAT2 for 24 h. The media was then replaced with DMEM media containing [U^13^C]-oleate (Sigma) in 5% dialyzed FBS (Sigma) and cells were incubated for 0, 4, 8 and 16 h (*n* = 3 per each time point). Dialyzed FBS was used to avoid confounders in the form of serum metabolites, thereby enriching the ‘tracer’ signal. While depleted of lipids, commercial dialyzed FBS contains growth factors and other nutrients so that it does not lead to cell growth arrest. At each time point cells were harvested and lipid lipids extracted using protocol described below. Chloroform and butanol extraction were both carried out to cover broader range of lipid classes. For chloroform extraction, cells were washed twice with ice-cold PBS and quenched by the addition of 0.75 mL ice cold methanol/PBS (1:1) on ice. The cells were then stored in −20 °C for 15 min. Thereafter, cells were scraped and transferred into pre-chilled 1.5 mL centrifuge tube, mixed with 50 µL 1 mM 3,5-di-tert-4-butylhydroxytoluene (BHT, Sigma), 1 µL SPLASH Lipidomix internal standard mix (Avanti Polar Lipids) per 1 × 10^5^ cells, 0.5 mL chloroform (Sigma). The mixture was vortexed vigorously for 1 min followed by centrifugation at 12,000 × *g* for 5 min. The lower chloroform layer was then collected into a fresh amber LC glass autosampler vial and dried under nitrogen gas. Samples were reconstituted at a concentration 20 µL per 1 mg of protein in chloroform/methanol (1:1, v/v) prior to the LC–MS analysis. For butanol extraction, cells were washed twice with ice-cold PBS. The cells were placed on ice and 0.6 mL ice-cold buffer containing 15 mM citric acid and 20 mM disodium hydrogen phosphate (pH 4.0) was added to the cells. The cells were then scraped into ice cold 2 mL centrifuge tube, mixed with 50 µL 1 mM BHT, 1 µL SPLASH Lipidomix internal standard mix per 2 × 10^5^ cells and 1 mL 1-butanol (Sigma). The mixture was vortexed vigorously for 10 min followed by centrifugation at 12,000 × *g* for 5 min. The upper butanol layer was then collected into a fresh amber LC glass autosampler vial, and butanol extraction was repeated by the addition of 0.5 mL butanol. The butanol fractions were combined and dried under nitrogen gas. Samples were reconstituted in 100 μL methanol (Sigma) prior to the LC–MS analysis.

### Liquid chromatography–mass spectrometry (LC–MS) analyses

Lipidomic analyses were performed using an Agilent 6560 ion mobility Q-TOF LC–MS coupled to a 1290 Infinity II UHPLC system. Chromatographic conditions were used as described previously^43^. For chloroform and butanol extracted lipids, 5 µL of sample was injected onto 1.7 µm particle 100 × 2.1 mm ID Waters Acquity CSH C18 column. Chloroform extracted lipids were separated using a gradient of mobile phase A (water/acetonitrile, 4:6, v/v) with 10 mM ammonium formate and mobile phase B (acetonitrile/2-propanol, 1:9, v/v) with 10 mM ammonium formate at a flow rate of 0.3 mL/min and column temperature maintained at 55 °C. The gradient ran from 0% to 40% mobile phase B from 0 to 6 min, increased from 40% to 100% mobile phase B over the next 24 min, maintained at 100% mobile phase B for another 4 min, and then returned to 0% mobile phase B over 2 min followed by 4 min to condition the column (40 min total). Lipids were analysed in positive ionization polarity mode. The electrospray settings were as follows: gas temperature 300 °C; drying gas flow 5 L/min; sheath gas temperature 300 °C; sheath gas flow 12 L/min; cap voltage 3.5 kV. For ‘unlabelled’ *T* = 0 h sample, data acquisition was performed using an Auto MS/MS mode, and for ^13^C-labelled samples data acquisition was performed using a full scan MS^1^ mode. The scan range was between 100 and 1700*m*/*z*. For Auto MS mode collision energy was 35 eV, 2 precursors per cycle and active exclusion for 0.5 min after 2 spectra. Mass correction was done by constant infusion of ‘lock mass’ ions (*m*/*z* 121.0509 and *m*/*z* 922.0098).

Butanol extracted lipids were separated using a gradient of mobile phase A (0.05% NH_4_OH in water) and mobile phase B (0.05% NH_4_OH in methanol) at a flow rate 0.2 mL/min and column temperature maintained at 50 °C. The gradient elution programme was as follows: 0–1 min isocratic 55% B, 1–14 min 55–85% B, 14–20 min 85–100% B, 20–22 min 100% B, 22–26 min return to 55% B followed by column equilibration for 4 min (30 min total). Lipids were analysed in negative ionization polarity mode with the same MS source temperature and voltage parameters as above. The scan range was between 100 and 1100 *m*/*z*. Mass correction was done by constant infusion of ‘lock mass’ ions (*m*/*z* 112.9856 and *m*/*z* 1033.9881).

### Lipidomic data processing and analysis

Peak detection and lipid identification was performed using Agilent Lipid Annotator 1.0. The list of identified lipids was converted into.csv file and used by MAVEN software^44^ as a library to analyse data acquired from stable isotope tracing experiment. Unlabelled and labelled intensities for each lipid were extracted, an unlabelled pool (*X*^U^) of each lipid class was calculated by summing up the intensities of unlabelled (*M* + 0) lipids, a labelled pool (*X*^L^) of each lipid class was calculated by summing up the intensities of ^13^C-oleate-containing lipids (*M* + 18, *M* + 36 and *M* + 54). Fluxes through individual lipid classes were calculated using kinetic flux profiling approach^45^, assuming cells were under metabolic steady state.

### Analyses of liver PA

Lipids were extracted from mouse liver slices following a modified Bligh and Dyer’s protocol as previously described^46^. The obtained lipidome was quantified using a targeted lipidomic approach^46^. Analyses of PA was conducted on a system comprising an Exion-UPLC coupled with a 6500 Plus QTRAP mass spectrometer (Sciex).

### Neutral lipid extraction

HeLa cells were grown in 10-cm dishes. 400 µM oleate was added to cells for 18 h when cells reached 80–90% confluence. A Triglyceride Assay Kit (Abcam) was used to determine the TAG levels according to the manufacturer’s protocol.

### Generation of the A2LKO mice

The Ethics Committee at Model Animal Research Center of Nanjing University approved all animal procedures used in this study, which comply with all relevant ethical regulations. Mice were housed under a light/dark cycle of 12 h with free access to food and water unless otherwise stated. Liver-specific AGPAT2 knock out mice in C57Bl/6J background were generated by the transgenic facility at Nanjing University in collaboration with GemPharmatech Co. The CRISPR/Cas9-based strategy used to disrupt *Agpat2* in mice is outlined in Fig. S7A: two Lox*P* sites were introduced into the *Agpat2* locus, sandwiching exons 2 and 4. Briefly, sgRNA was transcribed in vitro and donor vector was constructed accordingly. Cas9, sgRNA and donor were microinjected into the fertilized eggs of C57BL/6J mice. Fertilized eggs were then transplanted to obtain positive F0 mice which were confirmed by PCR and sequencing. A stable F1 generation mouse model was obtained by mating positive F0 generation mice with C57BL/6J mice. Homozygous *Agpat2*^fl/fl^ mice were then crossed with transgenic mice expressing *Cre* recombinase under the control of the albumin promoter. The resulting Agpat2^fl/+^Alb^−*cre*Tg/0^ progeny was then crossed with *Agpat2*^fl/fl^ mice to generate the A2LKO mice. Diet-induced obesity studies were carried out by feeding mice a HFD (60% calories from fat, Research Diets D12492) for 15 weeks at the age of 8 weeks.

### Triacylglycerol and cholesterol levels in liver

For Liver TAG levels, frozen liver chunks were saponified in ethanolic KOH, and the extracts were subsequently neutralized. The resultant free glycerol was determined with the Free Glycerol Reagent (F6428, Sigma-Aldrich) using glycerol (Sigma-Aldrich) as standard for calculation. For liver TC levels, lipids were extracted with chloroform:isopropanol:NP-40 (7:11:0.1), and TC was measured after removal of organic solvent using LabAssay Cholesterol kit (Wako Chemicals, USA).

### Primary mouse hepatocytes isolation and cell transfection

Primary mouse hepatocytes were isolated from 3-month-old mice using a collagenase-based method and cultured as previously described^47^. Hepatocytes from preparations with a cell viability ≥90% were seeded in six-well plates at a density of 1 × 10^6^ per well in a plating medium (M199 with GlutaMAXTM supplemented with 100 U/mL penicillin, 0.1 mg/mL streptomycin, 0.1% bovine serum albumin (BSA), 10% (v/v) foetal bovine serum, 10 nM insulin, 200 nM triiodothyronine, 500 nM dexamethasone) for 4 h and then incubated overnight (16 h) in a recovery medium (M199 with GlutaMAXTM supplemented with 100 U/mL penicillin, 0.1 mg/mL streptomycin and 10% (v/v) foetal bovine serum). The next morning, cells were used for transfection by plasmids with Lipofectamine-3000 (Thermo Fisher Scientific).

### Immunofluorescence staining and imaging

Primary hepatocytes were fixed by 4% paraformaldehyde, followed by permeabilization with 0.1% Triton X-100 in PBS for 10 min. The cells and the frozen tissue sections were stained by DAPI and BODIPY for 30 min. Coverslips were then mounted onto slides by using 50% glycerol in PBS. All images were obtained by Olympus FV1000 laser scanning confocal microscope (Olympus, Tokyo, Japan) with ×60 Plan Apochromat (1.4 NA) oil objective. Acquired images were quantified by ImageJ software.

### Primer information for qPCR analysis of expression of target genes

All primers and protocols used for expression analyses of selected mouse liver genes were from a previous study^11^, except CDS1 and CDS2 (Supplementary Table 3).

### CDS activity assay using mouse liver

CDS activity assay were carried out as described above except 100 µg liver tissues were used and the reaction was incubated at 30 °C for 30 min.

### Statistics and reproducibility

Data were analysed via *t*-test for two groups, or via one-way or two-way ANOVA for multiple groups using the Prism software (GraphPad, San Diego, CA, USA). Differences were considered statistically significant at *p* < 0.05.

### Reporting summary

Further information on research design is available in the Nature Research Reporting Summary linked to this article.

## Supplementary information

Supplementary Information

Reporting Summary

## Data Availability

A reporting summary for this article is available as Supplementary Information file. The main data supporting the findings of this study are available within the article and its Supplementary Figures. The exact *P* values for the data are also included within the Source Data file. Additional details on datasets and protocols that support the findings of this study will be made available by the corresponding author upon reasonable request. Source data are provided with this paper.

## Source data

Source Data
