# Supplementary material for: AGPAT2 interaction with CDP-diacylglycerol synthases promotes the flux of fatty acids through the CDP-diacylglycerol pathway
Source: Nat Commun. 2021 Nov 25;12:6877. doi: 10.1038/s41467-021-27279-4 (PMC8616899; doi:10.1038/s41467-021-27279-4)
Supplement: Supplementary file 1 — Supplementary Information [file 41467_2021_27279_MOESM1_ESM.pdf]

AGPAT2 interaction with CDP-diacylglycerol synthases promotes the flux of fatty acids through the CDP-diacylglycerol pathway

Mak *et al.*

Supplementary Information

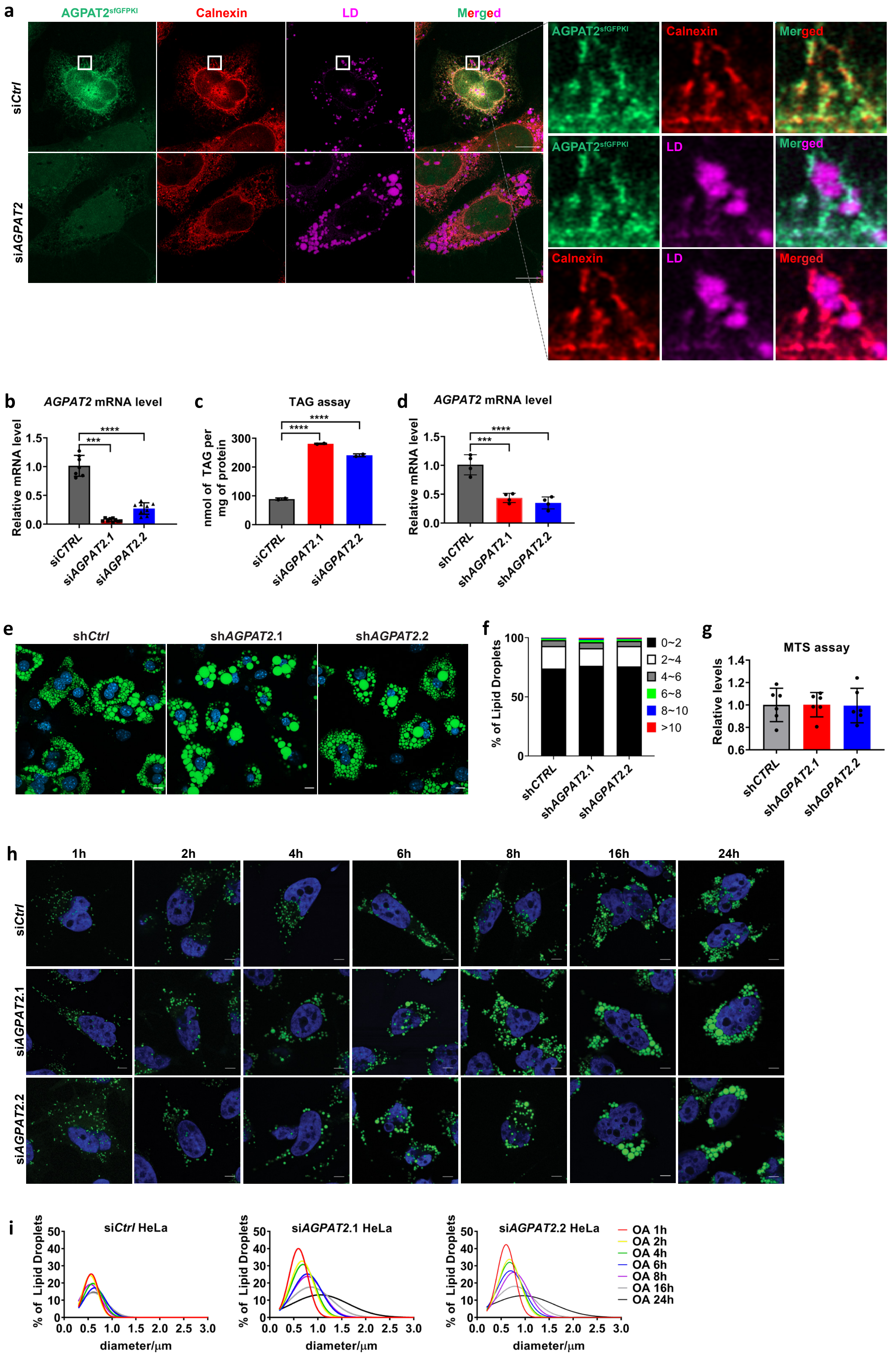
a
AGPAT2sfGFPKI
Calnexin
LD
Merged
siCtrl
siAGPAT2
b
AGPAT2 mRNA level
Relative mRNA level
siCTRL
siAGPAT2.1
siAGPAT2.2
c
TAG assay
nmol of TAG per mg of protein
d
AGPAT2 mRNA level
shCTRL
shAGPAT2.1
shAGPAT2.2
e
shCtrl
shAGPAT2.1
shAGPAT2.2
f
% of Lipid Droplets
0~2
2~4
4~6
6~8
8~10
>10
g
MTS assay
Relative levels
h
1h
2h
4h
6h
8h
16h
24h
i
siCtrl HeLa
siAGPAT2.1 HeLa
siAGPAT2.2 HeLa
% of Lipid Droplets
diameter/μm
OA 1h
OA 2h
OA 4h
OA 6h
OA 8h
OA 16h
OA 24h

Supplementary Figure 1

(a) Confocal imaging of fixed HeLa cells showing AGPAT2 tagged at its genomic locus with sfGFP. Cells were treated with control or *AGPAT2* siRNA, followed by 18 hours treatment of oleate (400µM). Bars=10 µm.

(b) *AGPAT2* siRNA knockdown efficiency. Cells were transfected with control or *AGPAT2* siRNA in HeLa cells. Cells were harvested 48 hours post transfection. *AGPAT2* mRNA levels of the transfected cells were examined using qRT-PCR. (mean ± SD; one-way ANOVA, ****p<0.0001, ***p<0.001, n=3 biologically independent experiments)

(c) TAG levels in control or *AGPAT2* knockdown HeLa cells with 8-hour oleate (100µM) treatment. (mean ± SD; one-way ANOVA, ****p<0.0001, n=3 biologically independent experiments)

(d) *AGPAT2* shRNA knockdown efficiency in 3T3-L1 adipocytes on day 8 of differentiation. 3T3-L1 adipocytes were transduced with lentivirus expressing Control or AGPAT2 shRNA, on day 6 of differentiation. (mean ± SD; one-way ANOVA, ****p<0.0001, n=3 biologically independent experiments)

(e) 3T3-L1 adipocytes were transduced with control or *AGPAT2* shRNA lentivirus on day 6 of differentiation for 24 hours. LDs were fixed and stained with BODIPY and visualized by confocal microscopy on day 8 of differentiation. Bars: 10 µm.

(f) Bar graphs show LD size distribution in 3T3-L1 adipocytes. Diameters of all LDs in a cell were measured and represented by red (>10 µm), blue (8-10 µm), green (6-8 µm), grey (4-6 µm), white (2-4 µm) and black (0-2 µm) (n=40-50 cells examined over 3 biologically independent experiments).

(g) Cell proliferation assay in *AGPAT2* knockdown 3T3-L1 adipocytes on day 8 of differentiation. (mean ± SD; one-way ANOVA, n=3 independent experiments)

(h) HeLa cells were transfected with control or *AGPAT2* siRNA for 24 hours, followed by the treatment with oleate (OA) at 400µM for indicated time points. LDs were stained with BODIPY and visualized by confocal microscopy. Bar=5µm. n=45-55 cells examined over 3 biologically independent experiments.

(i) Quantification of LDs diameters represented by Gaussian distribution.

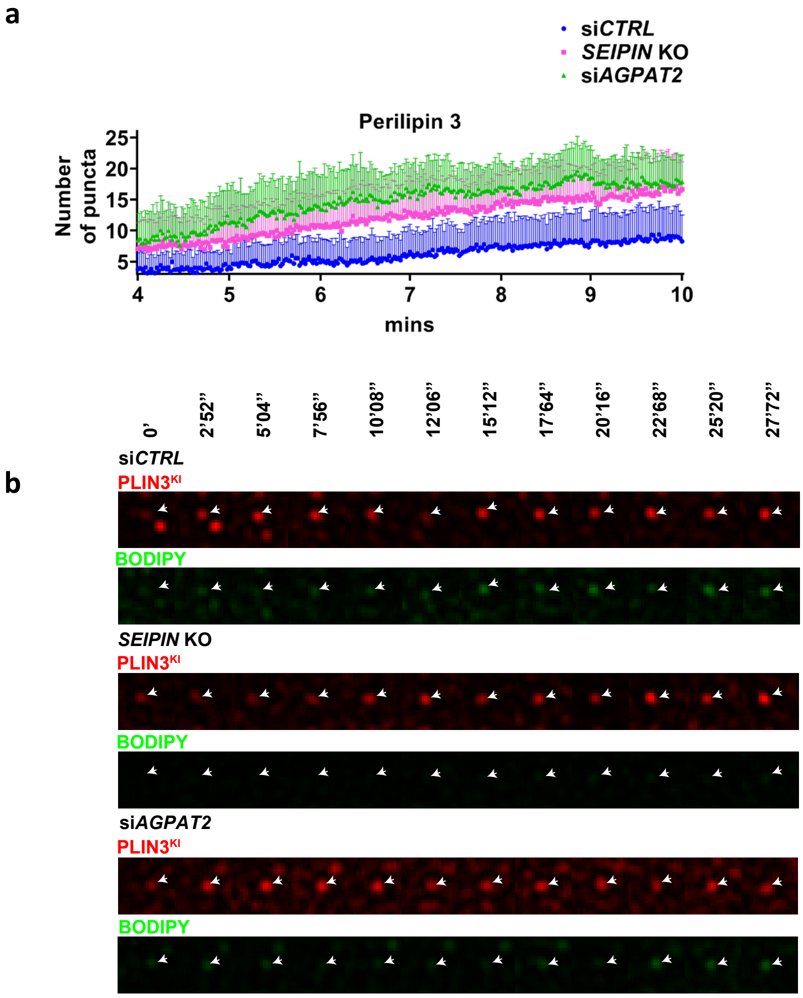

Supplementary Figure 2

(a) The number of PLIN3 puncta in control, *seipin KO* and *AGPAT2* knockdown HeLa cells from 4 to 10 minutes. (mean ± SD; Two-way ANOVA, n=15-20 cells examined over 3 biologically independent experiments)

(b) A representative gallery of images showing the accumulation of PLIN3 and BODIPY puncta over time in control, *SKO* and *AGPAT2* knockdown HeLa cells. White arrows indicate the newly formed PLIN3-positive LDs and their corresponding positions of BODIPY in each frame.

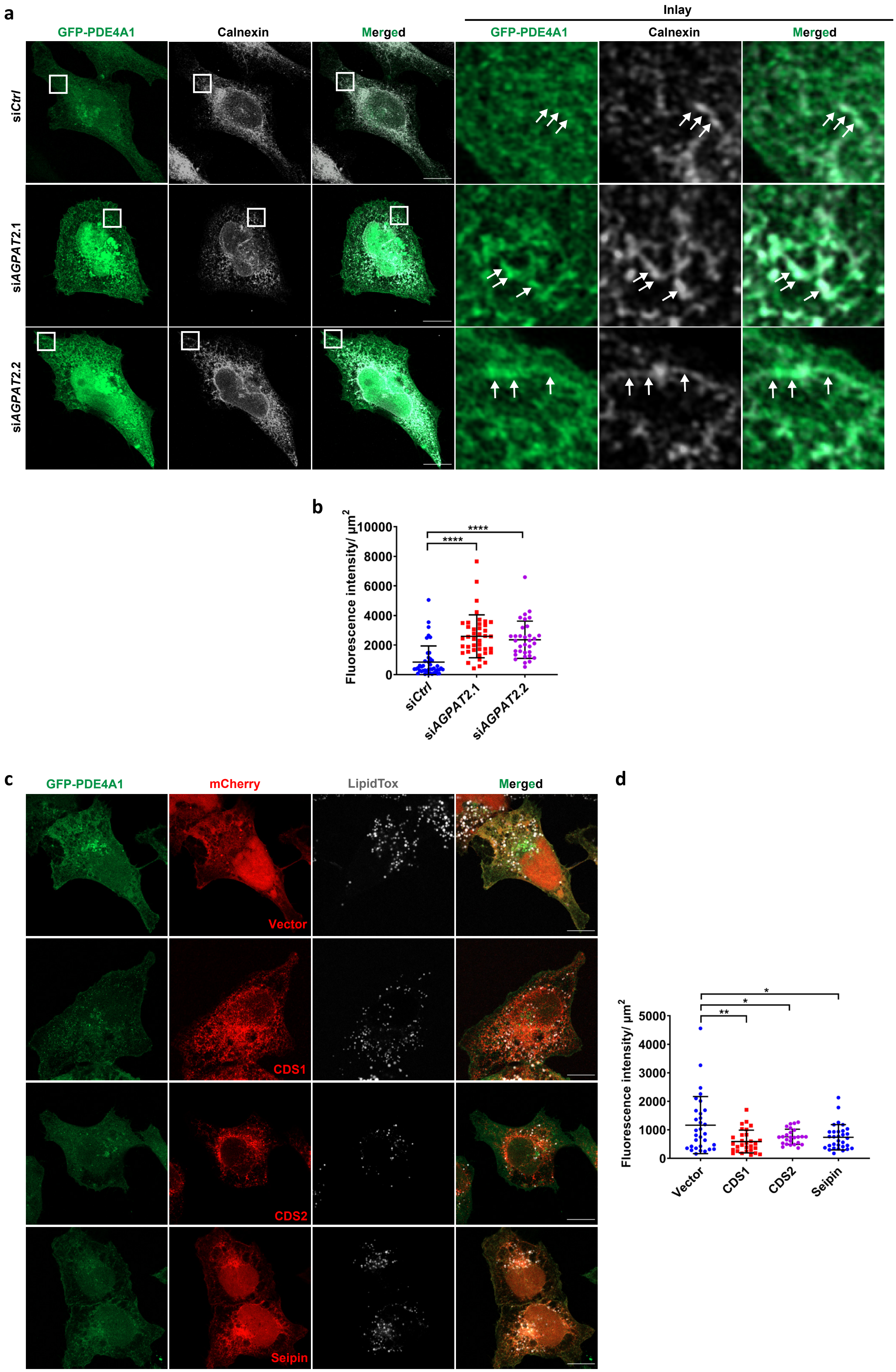
a
Inlay
GFP-PDE4A1
Calnexin
Merged
siCtrl
siAGPAT2.1
siAGPAT2.2
b
Fluorescence intensity/ µm2
10000
8000
6000
4000
2000
0
****
****
siCtrl
siAGPAT2.1
siAGPAT2.2
c
GFP-PDE4A1
mCherry
LipidTox
Merged
Vector
CDS1
CDS2
Seipin
d
Fluorescence intensity/ µm2
5000
4000
3000
2000
1000
0
*
*
**
Vector
CDS1
CDS2
Seipin

Supplementary Figure 3

(a) The fluorescence intensity of GFP-PDE4A1 in control and *AGPAT2* knockdown HeLa cells. Bars: 10 μm.

(b) Quantification of (a). Pixel intensity of whole cells were measured by ImageJ. (mean ± SD; one-way ANOVA, ****$p<0.0001$, n=20-30 cells examined over 3 biologically independent experiments)

(c) The fluorescence intensity of GFP-PDE4A1 in HeLa cells expressing hCDS1-mCherry, hCDS2-mCherry and mCherry-hSeipin. Bars: 10 μm.

(d) Quantification of (c). Pixel intensity of whole cells were measured by ImageJ. (mean ± SD; one-way ANOVA, **$p<0.01$, *$p<0.05$, n=20-30 cells examined over 3 biologically independent experiments)

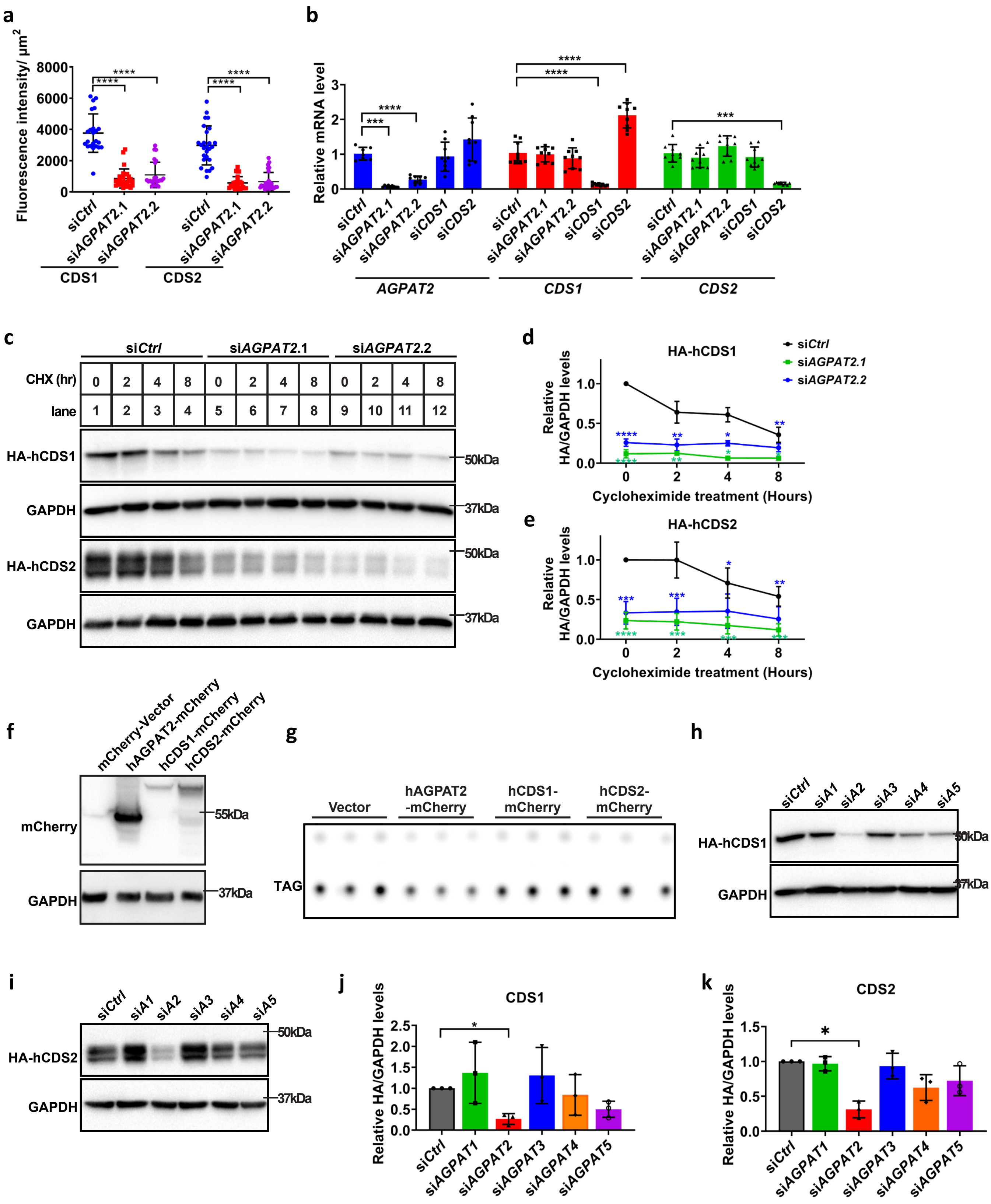

Supplementary Figure 4

(a) Quantification of total fluorescence intensity of HA as shown in Figure 4a. (mean ± SD; one-way ANOVA *p<0.05, **p<0.01, ***p<0.001, ****P < 0.0001, n= 23-28 cells examined over 3 biologically independent experiments)

(b) *AGPAT2, CDS1, CDS2* mRNA expression in control, *AGPAT2, CDS1 or CDS2* knockdown cells. Cells were harvested 48 hours post transfection. *AGPAT2* mRNA levels of the transfected cells were examined by qRT-PCR. (mean ± SD; two-way ANOVA, ****p<0.0001, ***p<0.001, n=3 biologically independent experiments)

(c) Immunoblot of HA-tagged CDS1/2 in Control, *AGPAT2* knockdown Huh7 cells treated with 20 µg/µl cycloheximide (CHX) for 0, 2, 4, 8 hours.

(d-e) Quantitation of relative band intensity of (c). (mean ± SD; two-way ANOVA, *p<0.05, **p<0.01, ***p<0.001, ****P < 0.0001, n=3 biologically independent experiments)

(f) Immunoblot of proteins for Figure 4g. n=3 biologically independent experiments.
(g) Raw image of Figure 4h.
(h-i) Immunoblot of HA-tagged CDS1/2 in AGPAT1-5 knockdown Huh7 cells.
(j-k) Quantification of band intensity of (h-i). (mean ± SD; one-way ANOVA ****$P < 0.0001$, n=3 biologically independent experiments).

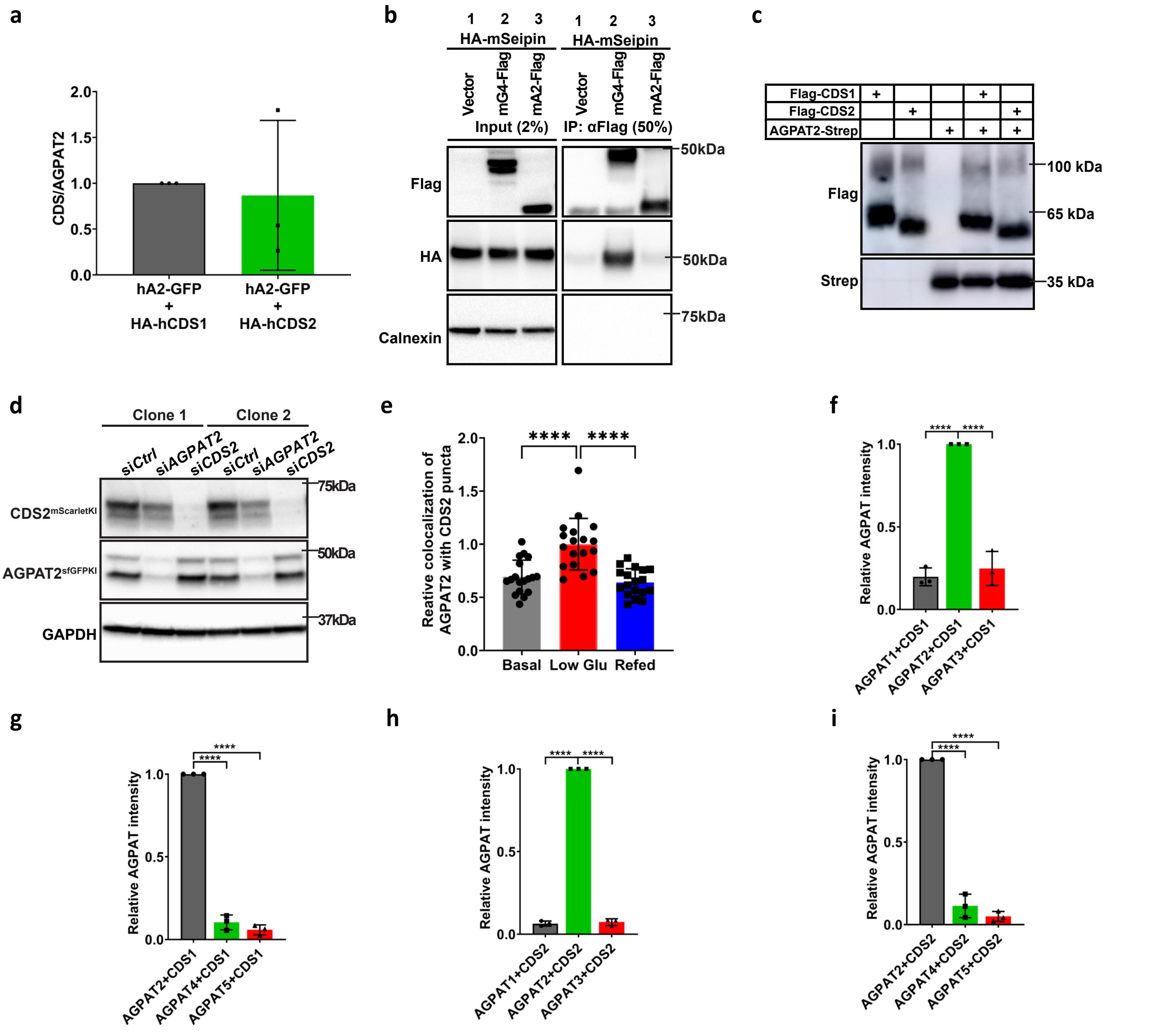

Supplementary Figure 5

(a) Quantitation of the levels of CDS1/2 relative to AGPAT2 as shown in Figure 5a. (mean ± SD; unpaired t-test, n=3 biologically independent experiments).

(b) Co-immunoprecipitation of Flag-tagged GPAT4/AGPAT2 and HA-tagged Seipin from transfected HEK293E lysates by anti-Flag antisera. n=3 biologically independent experiments.

(c) Immunoblot of Flag-tagged CDS1/2 and Strep-tagged AGPAT2 post purification/co-purification. n=3 biologically independent experiments.

(d) Immunoblot of endogenous AGPAT2-sfGFP and CDS2-mScarlet after siAGPAT2 or siCDS2 treatment. n=3 biologically independent experiments.

(e) Colocalization analysis of endogenously tagged AGPAT2-sfGFP and CDS2-mScarlet. Fluorescent intensity of 3 puncta from 6 cells were measured. (mean ± SD; one-way ANOVA, ****p<0.0001)

(f-i) Quantitation of Figure 5c-5f. (mean ± SD; one-way ANOVA ****P < 0.0001, n=3 biologically independent experiments)

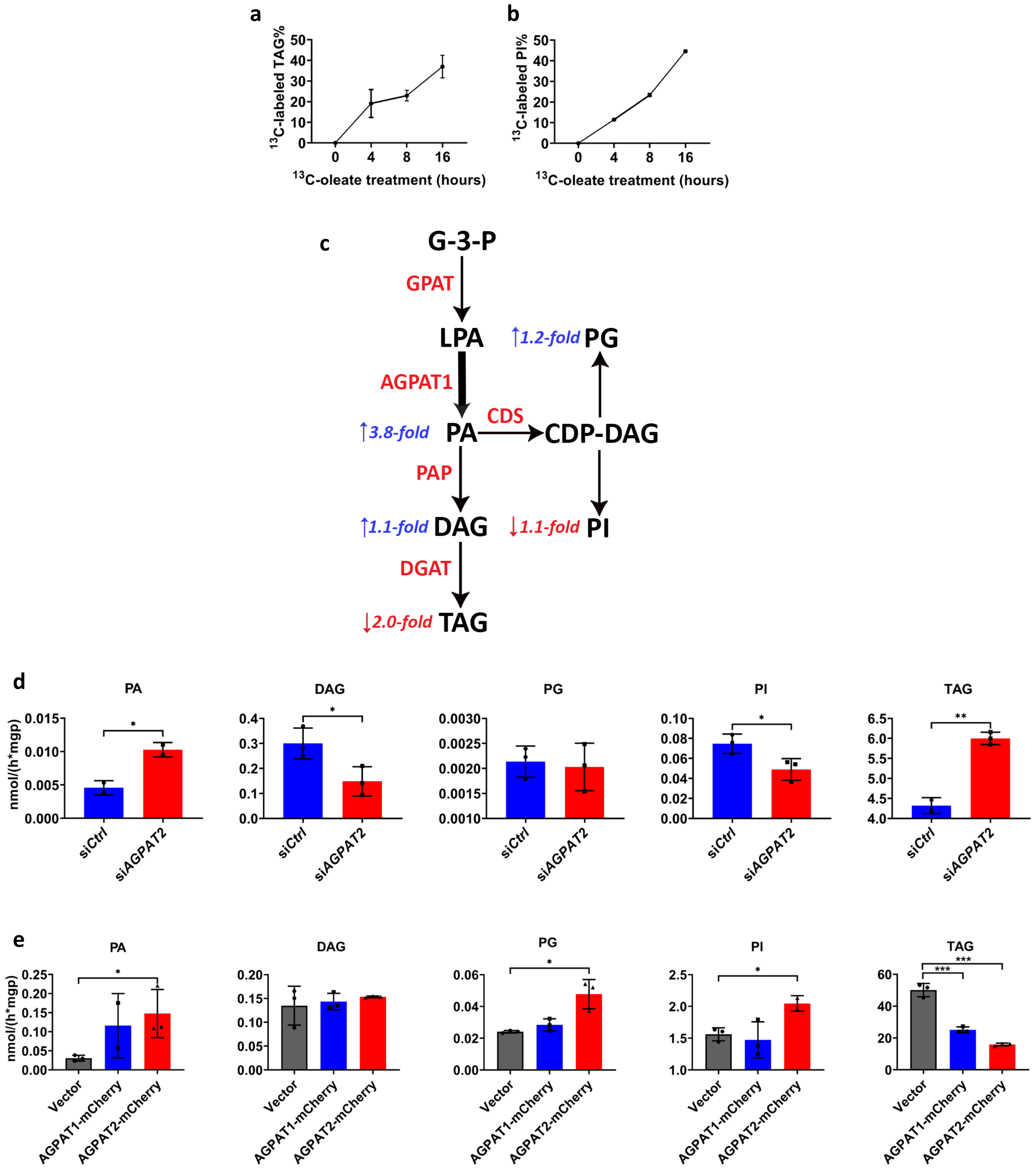

Supplementary Figure 6

(a-b) The fraction of 13C tracer incorporation in (a) TAG and (b) phosphatidylinositol lipid species after 0, 4, 8 and 16 hours of incubation. n=3 biologically independent experiments.

(c) HeLa cells were transfected with human AGPAT1-mCherry for 24 hours. Cells were then treated by dialysed FBS for 16 hr and then loaded for 8 hours with 10 µM [U13C]-oleate (C18:1). 13C-labelled samples were analysed by LC-MS. Fold changes of oleate incorporation were indicated as blue (increase) and red (decrease) arrows.

(d) The rate of oleate incorporation into PA, DAG, PI, PG and TAG in *AGPAT2* deficient HeLa cells as shown in Figure 6a. (mean ± SD; two-tailed unpaired t-test, ***p<0.001, **p<0.01, *p<0.05, n=3 biologically independent experiments).

(e) The rate of oleate incorporation into PA, DAG, PI, PG and TAG in AGPAT1/2-mCherry over-expressing HeLa cells as shown in Figure 6b and supplementary figure 6c. (mean ± SD; two-tailed unpaired t-test, ***p<0.001, **p<0.01, *p<0.05, n=3 biologically independent experiments).

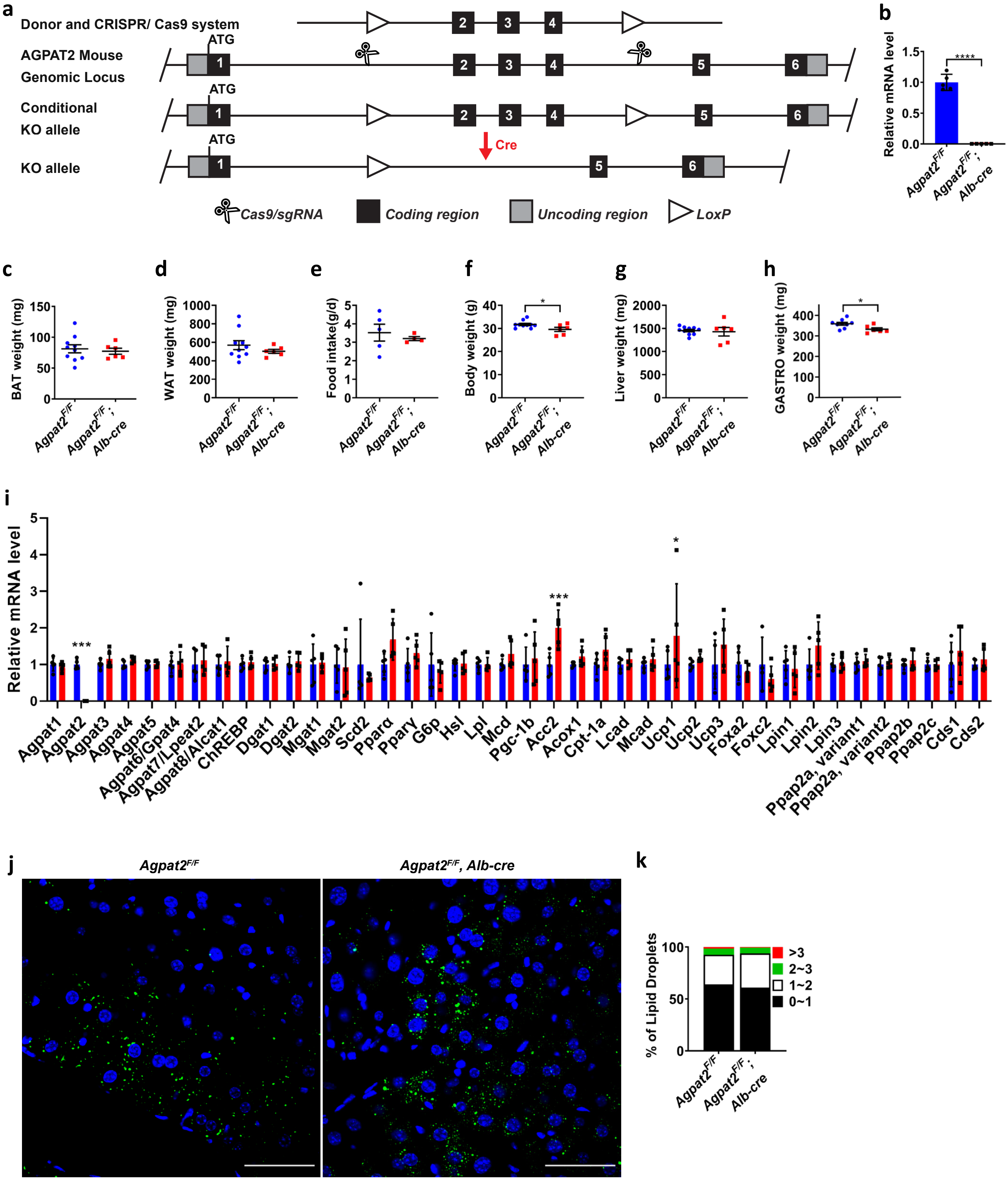

Supplementary Figure 7

(a) AGPAT2 disruption strategy by CRISPR/Cas9. **(b)** AGPAT2 mRNA in liver. WAT weight **(c)**, BAT weight **(d)**, food intake **(e)**, body weight **(f)**, liver weight **(g)**, gastro weight **(h)** of WT (Agpat2f/f) and A2LKO (Agpat2f/f, Alb-cre) mice fed chow. **(i)**, expression profiling of selected genes. b-i: WT, n=6-9; A2LKO, n=6-7. **(j)**, BODIPY-stained liver sections from WT and A2LKO mice fed chow. Bars: 50 µm. **(k)**, distribution of LDs of different diameters as shown in j. b to h, mean ± SD, *P < 0.05, ****P < 0.0001. i, mean ± SD; two-way ANOVA, *P < 0.05, ***P < 0.001.

Supplementary Table 1. Antibody, chemical and plasmids. Related to method section.

REAGENT OR RESOURCE	SOURCE	IDENTIFIER	DILUTION
**Antibodies**			
Rabbit polyclonal anti-HA	Cell Signaling Technology	Cat# 3724	1:1000 for western<br>1:1500 for IF
Rabbit monoclonal anti-GAPDH	Cell Signaling Technology	Cat# 5174	1:1000 for western
Rabbit monoclonal anti-AGPAT2	Cell Signaling Technology	Cat# 14937	1:1000 for western
Rabbit polyclonal anti-AGPAT2	ABclonal	Cat# A6518	1:1000 for western
Rabbit monoclonal anti-Calnexin	Cell Signaling Technology	Cat# 2679	1:1000 for western
Rabbit polyclonal anti-Calnexin	Proteintech	Cat# 10427-2-AP	1:200 for IF
Rabbit polyclonal anti-CDS2	Proteintech	Cat#13175-1-AP	1:500 for western
Rabbit polyclonal anti-CDS2	ABclonal	Cat# A16080	1:1000 for western
Mouse monoclonal anti-MTP	BD Transduction LaboratoriesTM	Cat# 612022	1:5000 for western
Mouse monoclonal anti-GFP	Santa Cruz Biotechnology	Cat# sc-9996	1:1000 for western
Rabbit polyclonal anti-mCherry	Abcam	Cat# ab167453	1:1000 for western
Mouse monoclonal Anti-Flag	Sangon Biotech	Cat# D191041	1:4000 for western
Mouse monoclonal Anti-Strep	Sangon Biotech	Cat# 191106	1:4000 for western
Mouse monoclonal anti-HA Agarose	Sigma-Aldrich	Cat# A2095	N/A
GFP-Trap Magnetic Agarose	ChromoTek	Cat# gtma-10	N/A
Peroxidase AffiniPure Donkey Anti-Rabbit IgG (H+L)	Jackson Immuno Research	Cat# JI711035152	1:5000 for western
Peroxidase AffiniPure Donkey Anti-Mouse IgG (H+L)	Jackson Immuno Research	Cat# 715-035-150	1:5000 for western

Goat anti-Rabbit IgG (H+L) Highly Cross-Adsorbed Secondary Antibody, Alexa Fluor Plus 647	Thermo Fisher Scientific	Cat# A32733	1:500 for IF
Goat anti-Rabbit IgG (H+L) Cross-Adsorbed Secondary Antibody, Alexa Fluor 594	Thermo Fisher Scientific	Cat# A-11012	1:500 for IF
**Bacterial and Virus Strains**			
Subcloning Efficiency DH5α Competent Cells	Thermo Fisher Scientific	Cat#18265017	
**Chemicals, Peptides, and Recombinant Proteins**			
BODIPY493/503	Thermo Fisher Scientific	Cat# D3922	
DMEM	Thermo Fisher Scientific	Cat# 21969035	
HCS LipidTOX Deep Red Neutral Lipid Stain	Thermo Fisher Scientific	Cat# H34477	
Lipofectamine™ RNAiMAX	Thermo Fisher Scientific	Cat# 13778150	
Lipofectamine™ LTX Reagent with PLUS™ Reagent	Thermo Fisher Scientific	Cat# 15338100	
Lipofectamine-3000	Thermo Fisher Scientific	Cat#	
FluoroBrite™ DMEM	Thermo Fisher Scientific	Cat# A1896701	
ProLong™ Live Antifade Reagent, for live cell imaging	Thermo Fisher Scientific	Cat# P36975	
ProLong® Antifade Gold Reagent with DAPI	Thermo Fisher Scientific	Cat# P36941	
ProLong® Antifade Gold Reagent	Thermo Fisher Scientific	Cat# P10144	
Puromycin	Thermo Fisher Scientific	Cat# A1113803	
TRIzol	Thermo Fisher Scientific	Cat# 15596026	
High-Capacity cDNA Reverse Transcription Kit	Thermo Fisher Scientific	Cat# 4368814	

Oleic Acid	Sigma-Aldrich	Cat# O1383-5G	
Bovine Serum Albumin (essentially fatty-acid free)	Sigma-Aldrich	Cat# A7030-50G	
Oleic Acid [1–13C]	Cambridge Isotope Laboratories, Inc.	Cat# CLM-460-PK	
KOD Hot Start DNA Polymerase	Merck Millipore	Cat# 71086	
Cytidine 5`-Triphosphate, [5-3H]-, Tetrasodium Salt,>97%	PerkinElmer	Cat# NET309001MC	
Oleic Acid [1–14C]	PerkinElmer	Cat# NEC317050UC	
Egg PA	Avanti polar Lipids, INC.	Cat# 840101	
16:0 Coenzyme A	Avanti polar Lipids, INC.	Cat# 870716	
Polyethyleneimine (PEI)	Polysciences	Cat# 24765-2	
GDN	Anatrace	Cat# GDN101	
Anti-Flag G1 Resin	GenScript	Cat# L00432	
Strep-Tactin Resin	IBA	Cat# 2-5030-025	
**Commercial Assay Kits**			
Triglyceride Assay Kit	Abcam	Cat# ab65336	
LabAssay Triglyceride kit	Wako Chemicals USA	Cat# 290-63701	
LabAssay Cholesterol kit	Wako Chemicals USA	Cat#294-65801	
**Experimental Models: Cell Lines**			
Human: HeLa	ATCC	Cat# CCL-2	
Human: HEK293E	ATCC	Cat# CRL-1573	
Mouse: AML12	ATCC	Cat# CRL-2254	

Human: Huh-7D12	CellBank Australia	Cat# 01042712	
Human: HEK293F	Thermo Fisher Scientific	Cat# R79007	
Human: LentiX-293T	TaKaRa<br><br>(A gift from Irina Voineagu)	Cat# 632180	
**Recombinant DNA**			
mCherry-hSeipin	(Fei et al., 2011)	N/A	
pBABE-HA-mSeipin	(Pagac et al., 2016)	N/A	
pBABE-mGPAT4-Flag	(Pagac et al., 2016)	N/A	
pCMV-hCDS1-mCherry	(Xu et al., 2019)	N/A	
pCMV-hCDS2-mCherry	(Xu et al., 2019)	N/A	
GFP-PDE4A1	(Kassas et al., 2017)	A gift from Nicolas Vitale	
pcDNA3.1-hLbCpf1(TYCV) (pY230)	(Gao et al., 2017)	Addgene Plasmid# 89355	
pSpCas9(BB)-2A-Puro (PX459) V2.0	(Ran et al., 2013)	Addgene Plasmid# 62988	
pMaCTag-P06	(Fueller et al., 2020)	Addgene Plasmid# 120017	
**siRNA**			
Human *AGPAT2.1* siRNA<br><br>5' CCUGUGUCAUCGUCUCCAA	This study	N/A	
Human *AGPAT2.2* siRNA<br><br>5' CGAGGGUACUCGCAACGAC	This study	N/A	
Human *DGAT1* siRNA<br><br>5’ GUUCCUGAAGGAUCCCUAUA	(Xu et al., 2019)	N/A	

Human *DGAT2* siRNA<br><br>5' AAGUGAAGUAGAGCACAGC	(Xu et al., 2019)	N/A	
Human *CDS1* siRNA<br><br>5' CUCACUUCCACCCUUUCUA	(Qi et al., 2016)<br><br>(Xu et al., 2019)	N/A	
Human *CDS1* siRNA<br><br>5' CUUGUUAUCCACAACCUAU	(Qi et al., 2016)<br><br>(Xu et al., 2019)	N/A	
**Software and Algorithms**			
ImageJ	National Institutes of Health	https://imagej.net; RRID: SCR_003070	
GraphPad Prism 8	GraphPad Software	https://www.graphpad.com/	
MAVEN	(Melamud, Vastag, & Rabinowitz, 2010)	http://maven.princeton.edu/	
MATLAB	*MABLAB software*	*https://au.mathworks.com/products/matlab.html*	

Supplementary Table 2. Cloning Primers. Related to method section.

**Plasmid**	**Cloning primers**
pCMV-AGPAT1-mCherryN1	F-5' AAAAAGCTAGCATGGACCCGTGGCC
	R-5' AAAAAACCGGTGGCTGGGCTGGCAAGAC
pCMV-AGPAT2-mCherryN1	F-5' AAAAAGCTAGCATGGGCCTGCTTGCC
	R-5' AAAAAACCGGTGGTTCCTTTTTCTTAAGCTCTTGG
pCMV-AGPAT3-mCherryN1	F-5' AAAAA GCTAGC ATGGACCTCATCGGGC
	R-5' AAAAAACCGGTGGGTCCGTTTGTTTCCGTTT
pCMV-AGPAT4-mCherryN1	F-5' AAAAA GCTAGC ATGCTGCTGTCCCTGG
	R-5' AAAAAACCGGTGGTGCTTTAATAACAAACCACAGG
pCMV-AGPAT5-mCherryN1	F-5' AAAAA TACGTA ATGGAGCTGTGGCCC

	R-5’ AGTCAGTCGACCTACTTGTACAGCTCGTCCATG
pBABE-puro -hAGPAT1-mCherry	F-5’ AAAAA TACGTA ATGGACCCGTGGCC
	R-5’ AGTCAGTCGACCTACTTGTACAGCTCGTCCATG
pBABE-puro -hAGPAT2-mCherry	F-5’ AAAAA TACGTA ATGGGCCTGCTTGCC
	R-5’ AGTCAGTCGACCTACTTGTACAGCTCGTCCATG
pBABE-puro-hAGPAT3-mCherry	F-5’ AAAAA TACGTA ATGGACCTCATCGGGC
	R-5’ AGTCAGTCGACCTACTTGTACAGCTCGTCCATG
pBABE-puro -hAGPAT4-mCherry	F-5’ AAAAA TACGTA ATGCTGCTGTCCCTGG
	R-5’ AGTCAGTCGACCTACTTGTACAGCTCGTCCATG
pBABE-puro -hAGPAT5-mCherry	F-5’ AGTCATACGTAATGTACCCATACGATGTTCCAGATTACGCTATGTTGGAGCTGAGGC
	R-5’ AGTCAGTCGACTTATACCTTCAAGGTGGGTT
pBABE-puro -HA-hCDS1	AGTCA TACGTA ATGTACCCATACGATGTTCCAGATTACGCTATGACAGAGCTGAGGCA
	AGTCA GTCGAC CTACTCGTCCTCTGTGGTG
pBABE-puro -HA-hCDS2	F-5’ CCCTGTGTCATCATCTCTAATGCCCAGAGCATCCTGGACATGATG
	R-5’ CATCATGTCCAGGATGCTCTGGGCATTAGAGATGATGACACAGGG
pBABE-puro-mAGPAT2-H98A-mCherry	F-5’ AGTCA TACGTA ATGTAC CCA TAC GAT GTT CCA GAT TAC GCT ATGGTCAACGACCCTC
	R-5’ AGTCA GTCGAC TCAGGAACTAGAGCAGGTG
pBABE-puro -HA-hSeipin	F-5’ AGTCATACGTAATGGATTACAAGGATGACGACGATAAGATGGACCCGTGGCCA
	R-5’ AGTCAGTCGACCTACTGGGCTGGCAAGACC
pBABE-puro-Flag-mAGPAT2	F-5’ AGTCATACGTAATGGATTACAAGGATGACGACGATAAGATGGACCCGTGGCCA
	R-5’ AGTCAGTCGACCTACTGGGCTGGCAAGACC
pCMV-hAGPAT2-GFP	F-5’AGTCAGCTAGCATGGAGCTGTGGCCG

	R-5' AGTCAACCGGTGGCTGGGCCGGCTGC
pCAG-Flag-hCDS1	F-5'AAATATGCGGCCGCATGTTGGAGCTGAGGCACCG
	R-5' CCGCTCGAGTTATACCTTCAAGGTGGGTTG
pCAG-Flag-hCDS2	F-5' AAATATGCGGCCGCATGACAGAGCTGAGGCAGAG
	R-5' CCGCTCGAGCTACTCGTCCTCTGTGGTGG
pCAG-hAGPAT2-Strep	F-5' AAATATGCGGCCGCATGGAGCTGTGGCCGTGTCTGGC
	R-5' CCGCTCGAGCTGGGCCGGCTGCACGCCAG
mCherry-mCDS2	F-5' AGTCACTCGAGGGATGACCGAACTACGGCA
	R-5' AGTCACCCGGGCTACTCATCTTCCAAGGCAGAT

Supplementary Table 3. RT-qPCR primer. Related to method section.

**Genes**	**qPCR primers (5' to 3')**	
Human-Agpat2	GTACTCTTCCTTCTCCTCCT	Forward
	TCTTGGAGATGTGGAGGAA	Reverse
Human-Cds1	CTGCCCAGTGGAATACCGAA	Forward
	AAGCTCACTCTTTCCTGTCTCA	Reverse
Human-CDS2	GGCTACAACGTCTACCACTC	Forward
	GAACCGGTGGTATTTACTGA	Reverse
Human-Actin	AGCGAGCATCCCCCAAAGTT	Forward
	GGGCACGAAGGCTCATCATT	Reverse
Mouse-Agpat1	GCTGGCTGGCAGGAATCAT	Forward
	GTCTGAGCCACCTCGGACAT	Reverse
Mouse-Agpat2	TTTGAGGTCAGCGGACAGAA	Forward
	AGGATGCTCTGGTGATTAGAGATGA	Reverse
Mouse-Agpat3	CCAGTGGCTTCACAAGCTGTAC	Forward
	CCCTGGGAATACACCCTTCTG	Reverse
Mouse-Agpat4	ACTTCGTGGAAATGATCTTTTGC	Forward

	GAGGTGCAGCAGGCTCTTG	Reverse
Mouse-Agpat5	AACGCAGGAACACCGATGTAT	Forward
	GAAAGGAGTTTTGTGTATGTTGCATT	Reverse
Mouse-Agpat6/Gpat4	CATGTCTGGTTTGAGCGTTCTG	Forward
	GGACATGCTCAGTCAGCCTCTT	Reverse
Mouse-Agpat7/Lpeat2	TGGCCTTTGAGCTCTTTGCT	Forward
	GGTGCTGAAGCCGTCTTTG	Reverse
Mouse-Agpat8/Alcat1	CCAGACAGCAGCCTCAAAAAC	Forward
	GATAGGGACCGAACACAGATCCT	Reverse
Mouse-ChREBP	CCTTCGCCAACTCAGCACTT	Forward
	TGGCTTGCTCAGGCACAA	Reverse
Mouse-Dgat1	GAGGCCTCTCTGCCCCTATG	Forward
	GCCCCTGGACAACACAGACT	Reverse
Mouse-Dgat2	CCGCAAAGGCTTTGTGAAG	Forward
	GGAATAAGTGGGAACCAGATCA	Reverse
Mouse-Mgat1	GAGTAACGGGCCGGTTTCA	Forward
	AGACATTGCCACCTCCATCCT	Reverse
Mouse-Mgat2	GCCCATCGAGGTGCAGAT	Forward
	AGAGCTCCTTGATATAGCGCTGAT	Reverse
Mouse-Scd2	CCACCCAGATGCTAAAATGATG	Forward
	TTCCGATTTTTGTCCGTTTTACA	Reverse
Mouse-Pparα	ACAAGGCCTCAGGGTACCA	Forward
	GCCGAAAGAAGCCCTTACAG	Reverse
Mouse-Pparγ	CACAATGCCATCAGGTTTGG	Forward
	GCTGGTCGATATCACTGGAGATC	Reverse
Mouse-G6p	TGGGCAAAATGGCAAGGA	Forward
	TCTGCCCCAGGAATCAAAAAT	Reverse
Mouse-Hsl	GGAGCACTACAAACGCAACGA	Forward
	TCGGCCACCGGTAAAGAG	Reverse

Mouse-Lpl	ACTCTGTGTCTAACTGCCACTTCAA	Forward
	ATACATTCCCGTTACCGTCCAT	Reverse
Mouse-Mcd	GGAGACAGGCCCCAACAGT	Forward
	TGAGGATCTGCTCGGAAGCT	Reverse
Mouse-Pgc-1b	CAGAAGCACGGTTTTATCACCTT	Forward
	GGGCTCATTGCGCTTTCTC	Reverse
Mouse-Acc2	GGGCTCCCTGGATGACAAC	Forward
	GCTCTTCCGGGAGGAGTTCT	Reverse
Mouse-Acox1	AGATTGGTAGAAATTGCTGCAAAA	Forward
	ACGCCACTTCCTTGCTCTTC	Reverse
Mouse-Cpt-1a	CACCAACGGGCTCATCTTCTA	Forward
	CAAAATGACCTAGCCTTCTATCGAA	Reverse
Mouse-Lcad	TCAATGGAAGCAAGGTGTTCA	Forward
	GCCACGACGATCACGAGAT	Reverse
Mouse-Mcad	GCAACTGCCCGCAAGTTT	Forward
	TACTCCCCGCTTTTGTCATATTC	Reverse
Mouse-Ucp1	GAGGTGTGGCAGTGTTCATTG	Forward
	GGCTTGCATTCTGACCTTCA	Reverse
Mouse-Ucp2	GCTTCTGCACCACCGTCAT	Forward
	GCCCAAGGCAGAGTTCATGT	Reverse
Mouse-Ucp3	TTTCTGCGTCTGGGAGCTT	Forward
	GGCCCTCTTCAGTTGCTCAT	Reverse
Mouse-Foxa2	ACTTTGGGAGAGCTTTGAGGAA	Forward
	CCCATCTATTTAGGGACACAGACA	Reverse
Mouse-Foxc2	AAAGCGCCCCTCTCTCAGA	Forward
	CTCAAACTGAGCTGCGGATAAGT	Reverse
Mouse-Lpin1	GAGCATGCCAAGACCAACATC	Forward
	CAATGGGAAGACGTGATCGA	Reverse
Mouse-Lpin2	GCATTAACCAAGCCACGTTGT	Forward

	AAAGGCGAGCACTGGTAGGA	Reverse
Mouse-Lpin3	GACTGCCTAAGGTGGCGAAAG	Forward
	CACAGATCGCCTCAGCCATA	Reverse
Mouse-Ppap2a, variant 1	TTTCTGTACTGACAACAGCGTGAA	Forward
	GTAAGCCAAGCCCCAGTATGG	Reverse
Mouse-Ppap2a,variant 2	CAAGGCATACCCCCTTCCA	Forward
	CCTAATAAGGCATAAGGTATGGTGTCT	Reverse
Mouse-Ppap2b	GGCACTTGCACATGTAGAAACC	Forward
	GGCATCCAGCAAGGATCTCA	Reverse
Mouse-Ppap2c	TGACTCCATCCGGTACCCATA	Forward
	TGATGACCCCAGCCATGAG	Reverse
Mouse-Cds1	CCCTCACCATGATCTCCCTC	Forward
	CTGCTCCTCCCTCTGAACAA	Reverse
Mouse-Cds2	GCTAGATGGAGAGACAGCGT	Forward
	CGATCATGGCCAAAGTCAGG	Reverse

Supplementary Table 4. Crispr Knock-in Primer. Related to method section.

F PLIN3 sgRNA human<br><br>CACCGCCCCGGAGGAGAAGAAGTAG
R PLIN3 sgRNA human<br><br>AAACCTACTTCTTCTCCTCCGGGGC
Human PLIN3 ssODN<br><br>GGACCACATGGTGGAATATGTGGCCCAGAACACACCTGTCACGTGGCTCGTGGGACCCTTTGCC<br>CCTGGAATCACTGAGAAAGCCCCGGAGGAGAAGAAGGGATCTGGATCTGGATCTGTGAGCAAG<br>GGCGAGGAGGATAACATGGCCATCATCAAGGAGTTCATGCGCTTCAAGGTGCACATGGAGGGC<br>TCCGTGAACGGCCACGAGTTCGAGATCGAGGGCGAGGGCGAGGGCCGCCCCTACGAGGGCACC<br>CAGACCGCCAAGCTGAAGGTGACCAAGGGTGGCCCCCTGCCCTTCGCCTGGGACATCCTGTCCC<br>CTCAGTTCATGTACGGCTCCAAGGCCTACGTGAAGCACCCCGCCGACATCCCCGACTACTTGAA<br>GCTGTCCTTCCCCGAGGGCTTCAAGTGGGAGCGCGTGATGAACTTCGAGGACGGCGGCGTGGTG<br>ACCGTGACCCAGGACTCCTCCCTGCAGGACGGCGAGTTCATCTACAAGGTGAAGCTGCGCGGCA

CCAACTTCCCCTCCGACGGCCCCGTAATGCAGAAGAAGACCATGGGCTGGGAGGCCTCCTCCGA<br>GCGGATGTACCCCGAGGACGGCGCCCTGAAGGGCGAGATCAAGCAGAGGCTGAAGCTGAAGG<br>ACGGCGGCCACTACGACGCTGAGGTCAAGACCACCTACAAGGCCAAGAAGCCCGTGCAGCTGC<br>CCGGCGCCTACAACGTCAACATCAAGTTGGACATCACCTCCCACAACGAGGACTACACCATCGT<br>GGAACAGTACGAACGCGCCGAGGGCCGCCACTCCACCGGCGGCATGGACGAGCTGTACAAGTG<br>AGGCGAGAGGAGAGGACTCAGCGGGCCCCGTCTCTATAATGCAGCTGTGCTCTGGAGTCCTCAA<br>CCCGGGGCTCATTTCAAACTTATTTCTAGCCAC
AGPAT2-F<br><br>CACCGGGCCATGAGGACCACCTTCCTCCACATCTCCAAGACCCCCCAGGAGAACGGGGCCACTG<br>CGGGGTCTGGCGTGCAGCCGGCCCAGTCAGGTGGAGGAGGTAGTG
AGPAT2-LbCpf1TYCV-R<br><br>TCCAGCCATCGGCTTCCACCTGCCCTCCCCAGGTCATGCCCTGCCGTGGTCTGGGAAAAAAGTG<br>CAGCCGGCCCAGTAGCCATCTACACTTAGTAGAAATTAGCTAGCTGCATCGGTACC
Human CDS2 ssODN<br><br>GGCGGGGCCGGCCGTGGGAGTCCGCGCGTGCCCGCGCCGAGCTGCCTGCTCCGGCGGCTTCGCT<br>GCTAGCTCGCGGCGACGTCGGGCCGATTTTCCCAGGATGGTGAGCAAGGGCGAGGCAGTGATC<br>AAGGAGTTCATGCGGTTCAAGGTGCACATGGAGGGCTCCATGAACGGCCACGAGTTCGAGATC<br>GAGGGCGAGGGCGAGGGCCGCCCCTACGAGGGCACCCAGACCGCCAAGCTGAAGGTGACCAA<br>GGGTGGCCCCCTGCCCTTCTCCTGGGACATCCTGTCCCCTCAGTTCATGTACGGCTCCAGGGCCT<br>TCACCAAGCACCCCGCCGACATCCCCGACTACTATAAGCAGTCCTTCCCCGAGGGCTTCAAGTG<br>GGAGCGCGTGATGAACTTCGAGGACGGCGGCGCCGTGACCGTGACCCAGGACACCTCCCTGGA<br>GGACGGCACCCTGATCTACAAGGTGAAGCTCCGCGGCACCAACTTCCCTCCTGACGGCCCCGTA<br>ATGCAGAAGAAGACAATGGGCTGGGAAGCGTCCACCGAGCGGTTGTACCCCGAGGACGGCGTG<br>CTGAAGGGCGACATTAAGATGGCCCTGCGCCTGAAGGACGGCGGCCGCTACCTGGCGGACTTC<br>AAGACCACCTACAAGGCCAAGAAGCCCGTGCAGATGCCCGGCGCCTACAACGTCGACCGCAAG<br>TTGGACATCACCTCCCACAACGAGGACTACACCGTGGTGGAACAGTACGAACGCTCCGAGGGC<br>CGCCACTCCACCGGCGGCATGGACGAGCTGTACAAGGGATCTGGATCTGGATCTATGACAGAG<br>CTGAGGCAGAGGGTGGCCCATGAGCCGGTTGCGCCACCCGAGGACAAGGTAGCGGCAGCGTCG<br>GGGTGGGCGCGGCCGGGACAGCGGCGCA
