# Supplementary material for: AGPAT2 interaction with CDP-diacylglycerol synthases promotes the flux of fatty acids through the CDP-diacylglycerol pathway
Source: Nat Commun. 2021 Nov 25;12:6877. doi: 10.1038/s41467-021-27279-4 (PMC8616899; doi:10.1038/s41467-021-27279-4)
Supplement: Supplementary file 2 — Reporting Summary [file 41467_2021_27279_MOESM2_ESM.pdf]

nature research

Corresponding author(s): Hongyuan Yang

Last updated by author(s): Oct. 1st, 2021

Reporting Summary

Nature Research wishes to improve the reproducibility of the work that we publish. This form provides structure for consistency and transparency in reporting. For further information on Nature Research policies, see our Editorial Policies and the Editorial Policy Checklist.

Statistics

For all statistical analyses, confirm that the following items are present in the figure legend, table legend, main text, or Methods section.

n/a	Confirmed	
☐	☒	The exact sample size (*n*) for each experimental group/condition, given as a discrete number and unit of measurement
☐	☒	A statement on whether measurements were taken from distinct samples or whether the same sample was measured repeatedly
☐	☒	The statistical test(s) used AND whether they are one- or two-sided<br>*Only common tests should be described solely by name; describe more complex techniques in the Methods section.*
☒	☐	A description of all covariates tested
☐	☒	A description of any assumptions or corrections, such as tests of normality and adjustment for multiple comparisons
☐	☒	A full description of the statistical parameters including central tendency (e.g. means) or other basic estimates (e.g. regression coefficient) AND variation (e.g. standard deviation) or associated estimates of uncertainty (e.g. confidence intervals)
☐	☒	For null hypothesis testing, the test statistic (e.g. *F*, *t*, *r*) with confidence intervals, effect sizes, degrees of freedom and *P* value noted<br>*Give P values as exact values whenever suitable.*
☒	☐	For Bayesian analysis, information on the choice of priors and Markov chain Monte Carlo settings
☒	☐	For hierarchical and complex designs, identification of the appropriate level for tests and full reporting of outcomes
☒	☐	Estimates of effect sizes (e.g. Cohen's *d*, Pearson's *r*), indicating how they were calculated

Our web collection on statistics for biologists contains articles on many of the points above.

Software and code

Policy information about availability of computer code

Data collection	Image Lab (Bio-rad, ver. 6.0.1)<br>Zeiss Zen software 3.1 .<br>Rotor-Gene Q (QIAGEN, Ver 2.3.5)<br>QuantaSmart™ Software (PerkinElmer)
Data analysis	ImageJ/ FIJI ver. 1.53m (https://imagej.net/software/fiji/downloads)<br>GraphPad Prism ver. 8 (https://www.graphpad.com/)<br>MAVEN ver. 6.2 (http://maven.princeton.edu/)<br>MATLAB Ver. R2020a (https://au.mathworks.com/products/matlab.html)

For manuscripts utilizing custom algorithms or software that are central to the research but not yet described in published literature, software must be made available to editors and reviewers. We strongly encourage code deposition in a community repository (e.g. GitHub). See the Nature Research guidelines for submitting code & software for further information.

Data

Policy information about availability of data

All manuscripts must include a data availability statement. This statement should provide the following information, where applicable:

- Accession codes, unique identifiers, or web links for publicly available datasets
- A list of figures that have associated raw data
- A description of any restrictions on data availability

All raw and processed data will be made available upon request.

Field-specific reporting

Please select the one below that is the best fit for your research. If you are not sure, read the appropriate sections before making your selection.

☒ Life sciences ☐ Behavioural & social sciences ☐ Ecological, evolutionary & environmental sciences

For a reference copy of the document with all sections, see nature.com/documents/nr-reporting-summary-flat.pdf

Life sciences study design

All studies must disclose on these points even when the disclosure is negative.

Sample size

No statistical methods were specifically used to calculate the sample size. Minimal numbers of animals, which could still generate statistically meaningful results, were used. Sample size was also determined based on the previous studies (Quan, Chao, et al, 2020; doi: 10.1038/s41467-020-16116-9.) and literature in the field using similar experimental paradigms and to reach a minimum of at least four mice per group.
For all other experiments, sample sizes were not pre-determined. We chose our sample size to give sufficient data values to conduct standard statistical tests. For all the in vitro experiments, n=3 was chosen as the minimal replicate number.
For the mice study, n=4-9 was used for WT (Agpat2f/f) and n=4-7 used used for liver specific AGPAT2 knockout (A2LKO/Agpat2f/f, Alb-cre) mice.
Data were analyzed via t-test for two groups, or via one-way or two-way ANOVA for multiple groups using Prism software (GraphPad, San Diego, CA, USA), and differences were considered statistically significant at $p < 0.05$.

Data exclusions

No samples or animals were excluded from the analyses.

Replication

All animal experiments and in vitro assays were repeated in at least three independent experiments. All replication attempts were successful.

Randomization

All samples/animals were randomly allocated to experimental groups and processed.

Blinding

The experiments were performed by four different groups. Each group analyzed the data individually before the first author compiles the figures together. Investigators were not blinded in cells and mice treatment. Blinding was performed in in vitro experiments. Determination of all parameters from cells and mice experiments are considered as objective measures, not subject to bias and therefore the integrity of the results are not impacted when running the study and analysis unblinded.

Reporting for specific materials, systems and methods

We require information from authors about some types of materials, experimental systems and methods used in many studies. Here, indicate whether each material, system or method listed is relevant to your study. If you are not sure if a list item applies to your research, read the appropriate section before selecting a response.

Materials & experimental systems

n/a	Involved in the study	
☐	☒	Antibodies
☐	☒	Eukaryotic cell lines
☒	☐	Palaeontology and archaeology
☐	☒	Animals and other organisms
☒	☐	Human research participants
☒	☐	Clinical data
☒	☐	Dual use research of concern

Methods

n/a	Involved in the study	
☒	☐	ChIP-seq
☒	☐	Flow cytometry
☒	☐	MRI-based neuroimaging

Antibodies

Antibodies used

- Rabbit polyclonal anti-HA (C29F4) (Cell Signaling Technology Cat# 3724)
- Rabbit monoclonal anti-GAPDH (D16H11) (Cell Signaling Technology Cat# 5174)
- Rabbit monoclonal anti-AGPAT2(D8W9B) (Cell Signaling Technology Cat# 14937)
- Rabbit polyclonal anti-AGPAT2 (ABclonal Cat# A6518)
- Rabbit monoclonal anti-Calnexin(C5C9) (Cell Signaling Technology Cat# 2679)
- Rabbit polyclonal anti-Calnexin (Proteintech Cat# 10427-2-AP)
- Rabbit polyclonal anti-CDS2 (Proteintech Cat#13175-1-AP)
- Rabbit polyclonal anti-CDS2 (ABclonal Cat# A16080)
- Mouse monoclonal anti-MTP (Clone 8) (BD Transduction LaboratoriesTM Cat# 612022)
- Mouse monoclonal anti-GFP (B-2) (Santa Cruz Biotechnology Cat# sc-9996)
- Rabbit polyclonal anti-mCherry (Abcam Cat# ab167453)
- Mouse monoclonal anti-HA Agarose (Clone HA-7) (Sigma-Aldrich Cat# A2095)
- GFP-Trap Magnetic Agarose (ChromoTek Cat# gtma-10)
- Peroxidase AffiniPure Donkey Anti-Rabbit IgG (H+L) (Jackson Immuno Research Cat#JI711035152)

• Peroxidase AffiniPure Donkey Anti-Mouse IgG (H+L) (Jackson Immuno Research Cat# 715-035-150)
• Goat anti-Rabbit IgG (H+L) Highly Cross-Adsorbed Secondary Antibody ,Alexa Fluor Plus 647 (Thermo Fisher Scientific Cat# A32733)
• Goat anti-Rabbit IgG (H+L) Cross-Adsorbed Secondary Antibody, Alexa Fluor 594 (Thermo Fisher Scientific Cat# A-11012)
• Mouse monoclonal Anti-Flag (Sangon Biotech Cat# D191041)
• Mouse monoclonal Anti-Strep (Sangon Biotech Cat# D191106)

Validation

• Anti-HA (CST Cat#3724) has been validated by CST by demonstrating immunoblotting on HeLa cells, untransfected or transfected with either HA-FoxO4 or HA-Akt3 (see website). This antibody was also cited by 886 publications (see website). HA-Tag (C29F4) Rabbit mAb detects exogenously expressed proteins containing the HA epitope tag. The antibody may cross-react with a protein of unknown origin ~100kDa. Species Reactivity: Human, Mouse, Rat, Hamster, Monkey, Virus, Mink, Chicken, Melanogaster, Xenopus, Zebrafish, Bovine, Dog, Pig, Cerevisiae, Elegans, Horse. Applications WB, IP, IHC, IF, Flow cytometry, Chromatin IP.
• Anti-GAPDH (CST Cat# 5174) has been validated by CST by demonstrating immunoblotting on HeLa, NIH3T3, C6 and COS-7 (see website). This antibody was also cited by 2230 publications (see website). GAPDH (D16H11) XP® Rabbit mAb detects endogenous levels of total GAPDH protein. Species Reactivity: Human, Mouse, Rat, Monkey. Applications WB, IHC, IF.
• Anti-AGPAT2 (CST Cat# 14937) has been validated by CST by demonstrating immunoblotting on 293, THP-1, MV-4-II and SK-MEL-5 (see website). AGPAT2 (D8W9B) Rabbit mAb recognizes endogenous levels of total AGPAT2 protein. Species Reactivity: Human. Applications WB, IP.
• Anti-AGPAT2 (ABclonal Cat# A6518) has been validated by ABclonal by demonstrating immunoblotting on HT-29 cells and Immunofluorescence analysis on HeLa cells (see website). Species Reactivity: Human, Mouse. Applications WB, IF.
• Anti-Calnexin (CST Cat# 2679) has been validated by CST by demonstrating immunoblotting on PANC1, HepG2 and A204 cells (see website). This antibody was also cited by 127 publications (see website). Calnexin (C5C9) Rabbit mAb detects endogenous levels of total calnexin protein. Species Reactivity: Human, Monkey. Applications WB, IHC, IF.
• Anti-Calnexin (Proteintech Cat# 10427-2-AP) has been validated by Proteintech by demonstrating Immunofluorescence on fixed HepG2 (website). This antibody was also cited by 84 publications (see website). Species Reactivity: Human, Hamster, Monkey, Mouse, Pig, Rat. Applications WB, IHC, IF, FC.
• Anti-CDS2 (Proteintech Cat#13175-1-AP) has been validated by Proteintech by demonstrating immunoblotting on Y79 cells (see website). This antibody was also cited by 2 publications (see website). Species Reactivity: Human, Zebrafish. Applications WB, IP, IF, ELISA.
• Anti-CDS2 (ABclonal Cat# A16080) has been validated by ABclonal by demonstrating immunoblotting on mouse brain, heart, kidney, and liver (see website). Species Reactivity: Human, Mouse. Applications WB, IHC.
• Anti-MTP (BD Transduction LaboratoriesTM Cat# 612022) has been validated by BD by demonstrating immunoblotting on mouse liver lysate (see website). This antibody was also cited by 6 publications (see website). Species Reactivity: Mouse, Rat. Applications WB, IHC, IP, ELISA.
• Anti-GFP (Santa Cruz Biotechnology Cat# sc-9996) has been validated by santa cruz by demonstrating immunoblotting on COS cells transfected with GFP fusion protein (see website). This antibody was also cited by 143 publications (see website). GFP (B-2) is recommended for detection of GFP and GFP mutant fusion Proteins. Species Reactivity: Species independent. Applications WB, IP, IF, FCM and ELISA.
• Anti-mCherry (Abcam Cat# ab167453) has been validated by Abcam by demonstrating immunoblotting on HEK293 cells transfected with pFin-EF1-mCherry vector (see website). This antibody was also cited by 204 publications (see website). Recombinant full length protein corresponding to mCherry. Species Reactivity: Species independent. Applications WB, ICC/IF.
• Mouse monoclonal anti-HA Agarose (Sigma-Aldrich Cat# A2095) was cited by 454 publications (see website). The antibody recognizes native as well as denatured-reduced forms of HA-tagged proteins and is reactive with N- or C-terminal HA-tagged fusion proteins expressed in E. coli or in mammalian cells. Suitable for use in immunoprecipitation assays and for immunoaffinity purification of HA-tagged fusion proteins.
• GFP-Trap Magnetic Agarose (ChromoTek Cat# gtma-10) has been validated by Chromotek by demonstrating immunoprecipitation of GFP-fusion proteins.
•Peroxidase AffiniPure Donkey Anti-Rabbit IgG (H+L) (Jackson Immuno Research Cat#JI711035152) was cited by 681 publications (see website). The antibody reacts with whole molecule rabbit IgG. It also reacts with the light chains of other rabbit immunoglobulins. No antibody was detected against non-immunoglobulin serum proteins. The antibody has been tested by ELISA and/or solid-phase adsorbed to ensure minimal cross-reaction with bovine, chicken, goat, guinea pig, syrian hamster, horse, human, mouse, rat and sheep serum proteins, but it may cross-react with immunoglobulins from other species. Minimal Cross Reactivity: Bovine, Chicken, Goat, Guinea Pig, Syrian Hamster, Horse, Human, Mouse, Rat, Sheep Serum Proteins
•Peroxidase AffiniPure Donkey Anti-Mouse IgG (H+L) (Jackson Immuno Research Cat# 715-035-150) was cited by 681 publications (see website). The antibody reacts with whole molecule mouse IgG. It also reacts with the light chains of other mouse immunoglobulins. No antibody was detected against non-immunoglobulin serum proteins. The antibody has been tested by ELISA and/or solid-phase adsorbed to ensure minimal cross-reaction with bovine, chicken, goat, guinea pig, syrian hamster, horse, human, rabbit and sheep serum proteins, but it may cross-react with immunoglobulins from other species. Minimal Cross Reactivity: Bovine, Chicken, Goat, Guinea Pig, Syrian Hamster, Horse, Human, Rabbit, Sheep Serum Proteins
• Goat anti-Rabbit IgG (H+L) Alexa Fluor Plus 647 (Thermo Fisher Scientific Cat# A32733) has been validated by Thermo Fisher by demonstrating immunofluorescence on A549 cells transfected with PMP70 (see website). This antibody was also cited by 189 publications (see website). Anti-Rabbit secondary antibodies are affinity-purified antibodies with well-characterized specificity for rabbit immunoglobulins and are useful in the detection, sorting or purification of its specified target. Species Reactivity: Rabbit. Applications WB, IHC, IHC-free, ICC/IF/ Flow cytometry, Misc.
• Goat anti-Rabbit IgG (H+L), AlexaFluor 594 (Thermo Fisher Scientific Cat# A-11012) has been validated by Thermo Fisher by demonstrating immunofluorescence on HeLa cells stained with alpha Tubulin Rabbit Polyclonal Antibody (see website). This antibody was also cited by 189 publications (see website). Anti-Rabbit secondary antibodies are affinity-purified antibodies with well-characterized specificity for rabbit immunoglobulins and are useful in the detection, sorting or purification of its specified target. Species Reactivity: Rabbit. Applications WB, IHC, IHC-free, ICC/IF/ Flow cytometry, Misc.
• Mouse monoclonal Anti-Flag (Sangon Biotech Cat# D191041) has been validated by Sangon Biotech by demonstrating immunoblotting on fusion proteins containing the Flag Tag (see website). This antibody recognizes the DYKDDDDK peptide fused to either the amino- or carboxy-terminus of targeted proteins. Species Reactivity: Species independent. Applications WB.
• Mouse monoclonal Anti-Strep (Sangon Biotech Cat# D191106) has been validated by Sangon Biotech by demonstrating immunoblotting on recombinant Strep tag II protein (see website). To also allow a Strep-tag to be placed at the N-terminus of recombinant proteins, it was re-engineered and re-named Strep-tag II. Species Reactivity: Species independent. Applications WB.

Eukaryotic cell lines

Policy information about cell lines

Cell line source(s)	HeLa (Human, ATCC-CCL-2)<br>HEK293E (Human, ATCC-CRL-1573)<br>AML12 (Mouse, ATCC-CRL-2254)<br>Huh-7D12 (Human, CellBank Australia, 01042712)<br>HEK293F (Human, Thermo Fisher Scientific, R79007)<br>3T3-L1 (Mouse, ATCC CL-173)<br>LentiX-293T (Human, TaKaRa, 632180)
Authentication	No further authentication of the cell lines was performed before use.
Mycoplasma contamination	The cell lines were tested negative for mycoplasma contamination by PCR analysis. We regularly treated the cells with mycoplasma removal agent (Bio-rad BUF035).
Commonly misidentified lines<br>(See ICLAC register)	No commonly misidentified cell lines were used.

Animals and other organisms

Policy information about studies involving animals; ARRIVE guidelines recommended for reporting animal research

Laboratory animals	Mice of C57Bl/6J background were used to generate AGPAT2f/f strain., then crossed with Albumin-Cre mice (Jackson laboratory, 003574). AGPAT2f/f (wild type) and AGPAT2f/f; Alb-cre (AGPAT2 liver specific knockout) were used in this study. Male and female mice of ~3-month old were used for analyses. The housing temperature and humidity of the mice were 21-25°C and 40-70% respectively.
Wild animals	This study did not involve wild animals.
Field-collected samples	This study did not involved samples collected from field.
Ethics oversight	The Ethics Committee at the Model Animal Research Center of Nanjing University, Nanjing, China, approved all animal procedures used in this study.

Note that full information on the approval of the study protocol must also be provided in the manuscript.
